# Michelson Interferometer for Global High-Resolution Thermospheric Imaging (MIGHTI) On-Orbit Wind Observations: Data Analysis and Instrument Performance

**DOI:** 10.1007/s11214-023-00971-1

**Published:** 2023-04-06

**Authors:** Christoph R. Englert, John M. Harlander, Kenneth D. Marr, Brian J. Harding, Jonathan J. Makela, Tori Fae, Charles M. Brown, M. Venkat Ratnam, S. Vijaya Bhaskara Rao, Thomas J. Immel

**Affiliations:** 1 Space Science Division, U.S. Naval Research Laboratory, Washington, DC 20375 USA; 2 Space Systems Research Corporation, Alexandria, VA 22314 USA; 3 Space Sciences Laboratory, University of California, Berkeley, Berkeley, CA 94720 USA; 4 Department of Electrical and Computer Engineering, University of Illinois Urbana-Champaign, Urbana, IL 61801 USA; 5 National Atmospheric Research Laboratory, Gadanki, Pakala, Andhra Pradesh 517112 India; 6 Department of Physics, Sri Venkateswara University, Tirupati, India

**Keywords:** ICON Explorer mission, Thermospheric wind, Limb sounding, Spatial Heterodyne Spectroscopy

## Abstract

The design, principles of operation, calibration, and data analysis approaches of the Michelson Interferometer for Global High-resolution Thermospheric Imaging (MIGHTI) on the NASA Ionospheric Connection (ICON) satellite have been documented prior to the ICON launch. Here we update and expand on the MIGHTI wind data analysis and discuss the on-orbit instrument performance. In particular, we show typical raw data and we describe key processing steps, including the correction of a “signal-intensity dependent phase shift,” which is necessitated by unexpected detector behavior. We describe a new zero-wind calibration approach that is preferred over the originally planned approach due to its higher precision. Similar to the original approach, the new approach is independent of any a priori data. A detailed update on the wind uncertainties is provided and compared to the mission requirements, showing that MIGHTI has met the ICON mission requirements. While MIGHTI observations are not required to produce absolute airglow brightness profiles, we describe a relative brightness profile product, which is included in the published data. We briefly review the spatial resolution of the MIGHTI wind data in addition to the data coverage and data gaps that occurred during the nominal mission. Finally, we include comparisons of the MIGHTI wind data with ground-based Fabry-Perot interferometer observations and meteor radar observations, updating previous studies with more recent data, again showing good agreement. The data processing steps covered in this work and all the derived wind data correspond to the MIGHTI data release Version 5 (v05).

## Introduction

### The MIGHTI Instrument

The Michelson Interferometer for Global High-resolution Thermospheric Imaging (MIGHTI) instrument onboard the NASA Ionospheric Connection (ICON) Explorer mission has now provided thermospheric wind and temperature measurements for about 3 years. The wind velocity observation is based on the Doppler shift measurement of the forbidden atomic oxygen red and green lines at the wavelengths of 630.0 nm (O(^1^D$\rightarrow ^{3}$P)) and 557.7 nm (O(^1^S$\rightarrow ^{1}$D)) respectively, imaging the limb of the Earth between 90 and 300 kilometers tangent point altitude during day and night. Wind directions are determined by combining observations of two fields of view that are directed nominally at an azimuth angle of 45 degrees and 135 degrees from the spacecraft ram direction, each providing line of sight wind components. For the wind observations, MIGHTI uses the Doppler Asymmetric Spatial Heterodyne (DASH) technique (Englert et al. 2007, 2017a). Details on the instrument design and wind data analysis can be found in publications by Englert et al. (2017a), Harlander et al. (2017), and Harding et al. (2017a). Here we update and expand on the MIGHTI wind data analysis and discuss the on-orbit instrument performance. We start with introductory remarks about the data coverage and continue in Sect. 2 with the characteristics of the raw on-orbit data.

Section 3 explains in detail all the critical data processing steps and quality checks that are taken to operationally retrieve vertical profiles of the horizontal thermospheric wind from the MIGHTI raw data. Most of the steps were already introduced by Englert et al. (2017a), but here we include illustrative on-orbit data and some additional processing steps that were found to be necessary after inspection of the on-orbit telemetry. In particular, the checks and processing steps described here include:

- Stray light assessment
- Dark current removal
- Spike correction
- Flatfielding (Responsivity and fringe modulation efficiency)
- Phase distortion correction
- Thermal drift (Interferometer drift and notch drift)
- Star calibration (pointing verification)
- Sufficient signal verification
- Low-signal phase shift
- Zero-wind phase
- Cardinal wind retrieval
- Quality flags

Section 4 discusses the wind data uncertainties. Section 5 shows the comparison of the on-orbit performance with the mission requirements. Section 6 presents inter-comparisons of the MIGHTI green- and red-line wind observations and comparisons of the MIGHTI wind data with coincident, ground-based observations from Fabry-Perot interferometers and meteor radars. Section 7 briefly covers the spatial resolution of the wind observations, and Sect. 8 describes the determination of the airglow’s relative volume emission rate for the oxygen red- and green-lines. Finally, Sect. 9 summarizes the major conclusions.

### MIGHTI Data Coverage

**Fig. 1:**
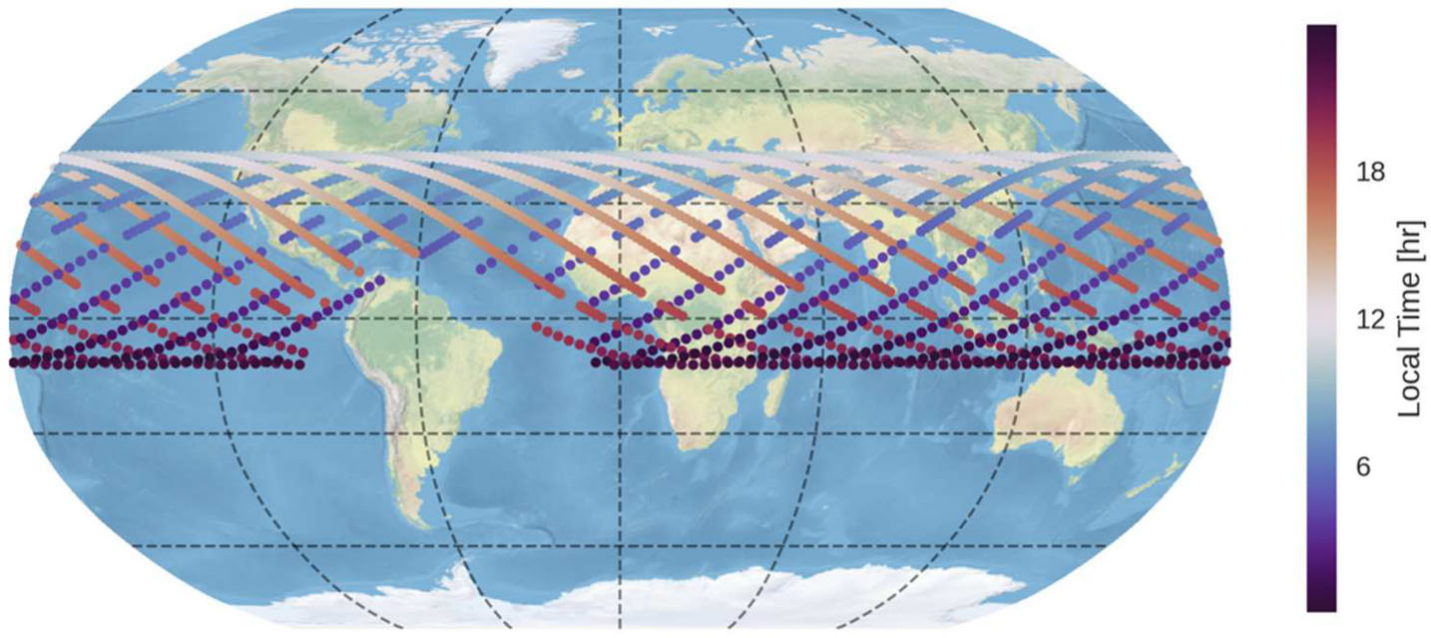
Geographic coverage of the MIGHTI observations for one day (08 May 2020). The gaps in the tracks are predominantly due to day/night mode changes and the South Atlantic Anomaly (SAA). Every color-coded dot represents an observed wind profile, taken every 30 seconds and every 60 seconds during day and night, respectively. The colors represent the local solar time of the observation. Note that the precession rate of the ICON satellite is approximately 30 minutes per day

The geographic coverage of the MIGHTI data is determined by the inclination of the ICON orbit and the MIGHTI viewing geometry. During normal science operations of the nominal mission, the two MIGHTI fields of view were almost exclusively pointed at the earth’s limb, 45° and 135° from the satellite ram direction, respectively, toward the northern hemisphere. Combining the resulting, nearly coincident observations with virtually perpendicular viewing directions allows for the determination of vertical profiles of horizontal wind vectors (Englert et al. 2017a; Harding et al. 2017a). Considering ICON’s nearly circular orbit altitude of approximately 600 km and the inclination of 27°, the latitudinal coverage is between about 12° South and 42° North, as shown in Fig. 1.

The local time coverage of the MIGHTI observations is also governed by the orbit. As is typical for a low inclination, low-Earth orbit, ICON flies through all local times during every orbit. However, in order to obtain full local time coverage for specific latitudes, one has to rely on the orbital precession. With a full precession period of about 48 days (Immel et al. 2023), it takes this long for the ascending orbit equator crossing to move through 24 hours of local time. Considering that the descending equator crossing of the ICON satellite is 12 hours out of phase with the ascending equator crossing and the fact that the MIGHTI observations are displaced by about 1700 km (great circle arc) from the satellite’s ground track toward the northern hemisphere, full local time coverage using both ascending and descending nodes can be achieved in half the precession period for the latitude band around approximately 13.5°. However, for the lowest and highest latitude regions covered by MIGHTI, 12° South and 42° North, respectively, the full precession period of about 48 days is needed to achieve full local time coverage.

**Fig. 2:**
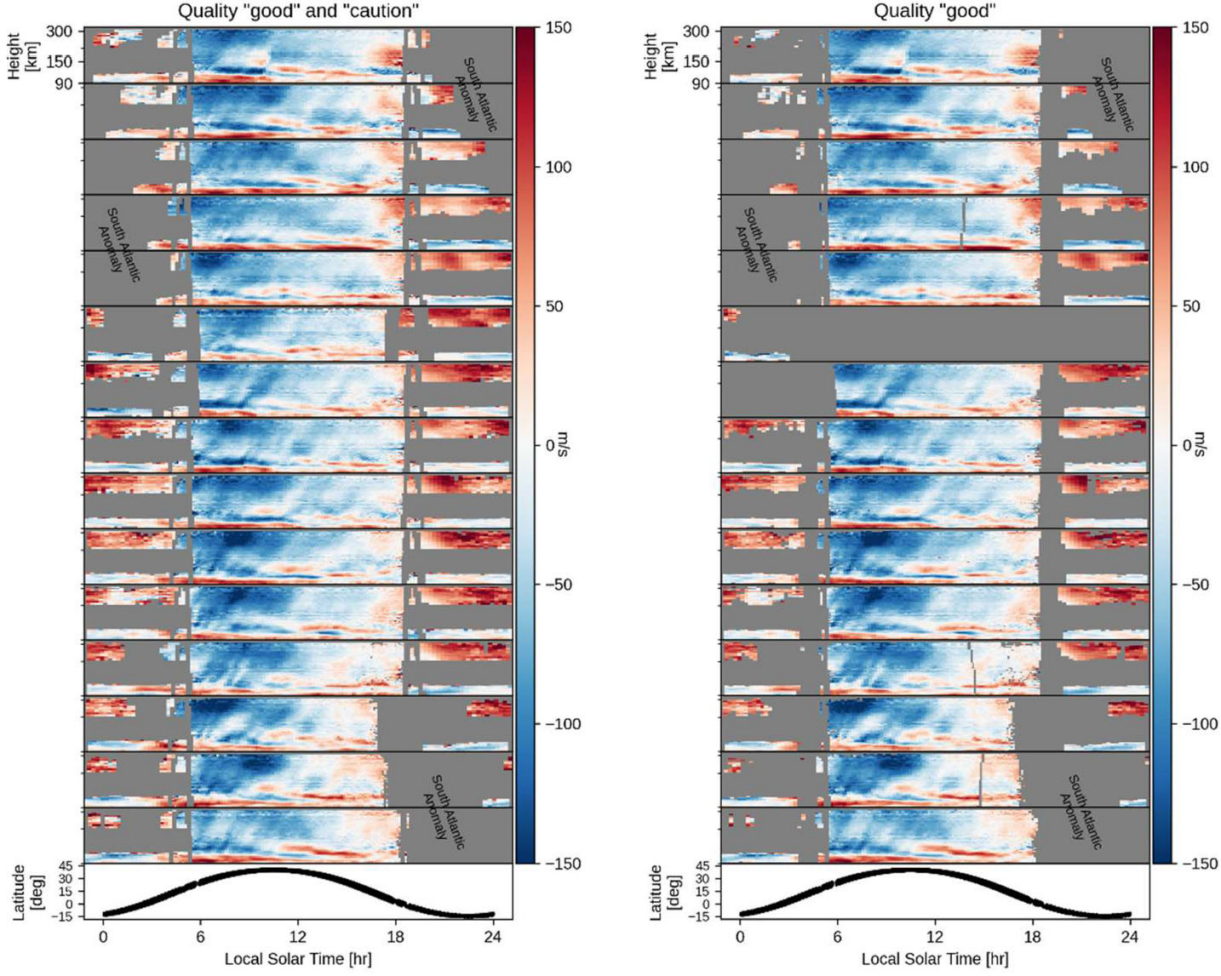
The left panel shows one day of MIGHTI wind data (08 May 2020), including data of quality “good” and “caution” (see Sect. 3.13). The right panel shows one day of MIGHTI wind data, including data of quality “good” only. Missing data is shown in gray. Red and blue shades represent eastward and westward winds, respectively

Figure 2 shows the MIGHTI zonal winds retrieved from the 15 orbits of one day in May 2020. Each sub-panel represents one orbit and the lowest panel illustrates the latitude/local-time relation. The left panel shows one day of MIGHTI wind data, including data of quality “good” and “caution” (see Sect. 3.13.). The right panel shows one day of MIGHTI wind data, including data of quality “good” only. Missing data is shown in gray. Missing data is predominantly due to (1) the South Atlantic Anomaly, in which the increased radiation environment at the detectors causes too much interference to retrieve robust wind speeds, (2) observations with too little airglow signal to retrieve robust wind speeds, and (3) day/night mode changes of the instrument, which occur close to the terminators. Note that even though for the data shown in Fig. 2, the data affected by the SAA is almost exclusively at night, these data outages progress in local solar time according to the approximately 30-minute per day local time precession of the ICON orbit. The missing orbit in the right plot is the calibration orbit wherein the calibration lamps are on. Although the algorithm is designed to minimize the influence of signal from the calibration lamps, and there is no evidence of systematic differences between lamp-on and lamp-off data, we have chosen for the sake of conservatism to mark this entire orbit as “caution.”

## Raw Data Characteristics

In preparation for the in-depth discussion of the MIGHTI wind data analysis in the next section, this section briefly reviews the high-level optical design of the two identical MIGHTI sensor units (MIGHTI-A and MIGHTI-B), shows examples of on-orbit data, and introduces the main effects that determine the phase of the observed interference fringes, for both oxygen red line ($\lambda =630$ nm) and green line ($\lambda =557.7$ nm) emissions, which are used to determine the vertical profiles of the horizontal thermospheric wind.

**Fig. 3:**
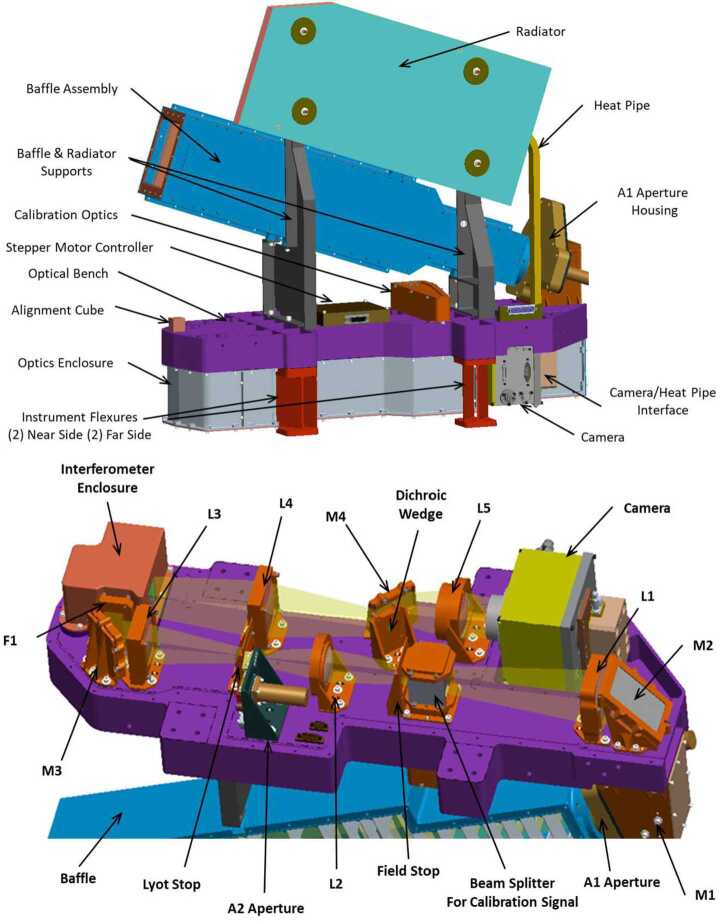
Top: View of one MIGHTI sensor unit. Bottom: Set-up of MIGHTI optical bench (purple) populated with all optical elements. L denotes lenses, M denotes mirrors, F denotes interference filters (Englert et al. 2017a). Light enters the sensor at the front of the baffle (top left in the image on the left and bottom left in the image on the right)

Figure 3 shows the overall optical set-up of the MIGHTI sensors. Light from the earth’s limb enters the baffle (top left in the top panel of Fig. 3). A simple two-mirror periscope (M1, M2) folds the beam onto the optical bench, where the limb scene is imaged on the interferometer gratings and then relayed onto the camera focal plane array (FPA). Between M2 and the interferometer, signal from quasi-monochromatic neon ($\lambda =630.48$ nm) and krypton ($\lambda =557.03$ nm) on-board calibration lamps can be superimposed onto the atmospheric signal via a 95%/5% beamsplitter. The etendue of the instrument can be reduced by approximately 85% by partially closing aperture A2 for the observation of the bright daytime airglow. After the interferometer modulates the signal from the limb scene with a wavelength-dependent fringe pattern, the signal is spatially separated into the “green” and “red/infrared” images by a dichroic wedge, so the different spectral components can be detected separately on different areas of the FPA. A detailed review of the optical elements and their specifications was previously published by Englert et al. (2017a).

**Fig. 4:**
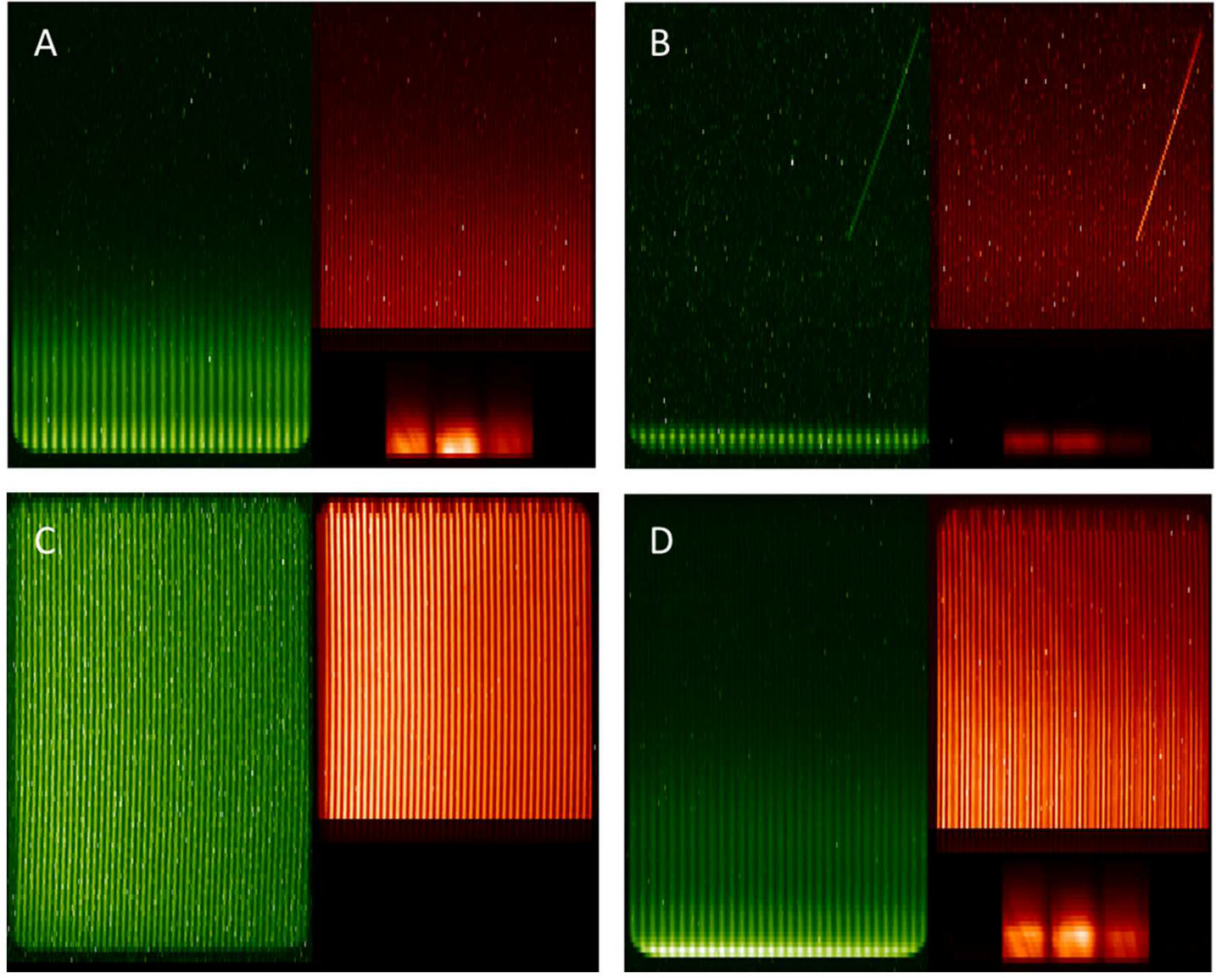
Example on-orbit exposures. (**A**) MIGHTI-A, daytime, atmosphere signal only; (**B**) MIGHTI-A, nighttime, atmosphere signal only, with signature of a bright star; (**C**) MIGHTI-B, calibration signal only; (**D**) MIGHTI-A, daytime, atmosphere and calibration signals. The grating registration marks, or notches, are easy to recognize on the top edge of the images in panels C and D

Figure 4 shows four typical, raw MIGHTI exposures of the Earth’s limb in false color. The vertical dimension of the images is equivalent to tangent height in the atmosphere, ranging from approximately 90 km on the bottom to 300 km on the top. The horizontal dimension is equivalent to the optical path difference in the interferometer, which produces the fringe modulation. The observed limb scene is imaged onto the FPA in both dimensions, so that features within the scene, such as stars, are in focus at the FPA. Panel A shows a daytime image with an integration time of 30 seconds, containing only airglow signal. On the left and top right of panel A, are the signals from the atomic oxygen green and red airglow lines, respectively. On the bottom right are five separate filter areas for the multi-spectral measurement of the molecular oxygen A-band and the associated background (Stevens et al. 2022). Panel B shows a typical nighttime exposure, with the narrow nighttime lower-thermospheric green line layer and a bright star within the field of view. The star’s signal creates a line, due to the change in MIGHTI attitude with respect to the inertial frame during the nighttime 60-second integration. Panel C shows an exposure for which aperture A1 blocks all light from the atmosphere, and the calibration lamps are turned on. The illumination of the detector areas for the oxygen green and red lines is nearly homogeneous, revealing the grating registration marks, or notches, on the top edge of the images. Panel D shows a daytime exposure containing both airglow and calibration signals. Especially for the red signal, one can easily see the beat-pattern of the two superimposed fringe patterns with different spatial frequencies. The dynamic range of the color scales in each image is not the same, but adjusted to avoid saturation. The speckles seen in all four images of Fig. 4 that are confined to one or a few binned pixels are due to hot pixels of the FPA, or are from signals generated by cosmic rays that pass through the FPA during the exposure.

The information on the Doppler wind speed for a given tangent point height is contained in the phase of the interference fringe at the corresponding FPA row (Englert et al. 2007, 2017a; Harding et al. 2017a). However, instrumental effects such as thermal drifts can also shift the fringe phase. Therefore, great care must be taken to isolate the wind speed information in the observed phase shift.

The Doppler shift of the airglow signal results in only a very small change of the phase. For the red line it is about 1.8 mrad for every 1 m/s in wind speed, or one thousandths of a full fringe for every 3.5 m/s. Therefore, small thermal changes in the instrument, which can result in phase changes of similar magnitude have to be monitored and removed during data processing. The following Table 1 provides a quick reference on what MIGHTI-A fringe phase shifts, in radians or in detector pixel units, are equivalent to a Doppler shift caused by a speed of 1 m/s. The numbers for MIGHTI-B are similar. Note that the given phase shifts in units of mrad are slightly different from the ones that can be derived from the information given by Englert et al. (2017a) before launch. This is because the interferometer optical path difference was updated with an improved optical analysis, using the accurate, as-built dimensions.

**Table 1:** MIGHTI-A fringe phase shift equivalents, corresponding to a Doppler shift caused by a speed of 1 m/s

MIGHTI A	Phase shift equivalent to a 1 m/s Doppler shift	
	Phase Shift [mrad]	Phase shift [binned horizontal pixels]
Oxygen green line	2.10	6.68 × 10^−3^
Krypton cal line	2.10	3.18 × 10^−3^
Oxygen red line	1.80	2.48 × 10^−3^
Neon cal line	1.80	3.22 × 10^−3^

There are two main thermal effects that have to be considered. The first is the drift of the interferometer that is due to thermal changes of the optical path difference inside the interferometer. Over small wavelength ranges, the interferometer drift can be considered as independent of fringe frequency and is therefore the same for atmospheric and calibration lines. Thus, it can be removed directly using the observed shift of the calibration line fringes. The second source of possible drift is the mechanical distortion of the instrument which can result in a lateral shift of the grating image on the FPA. This is equivalent to a phase shift, as the fringe phase is referenced to the FPA pixels. This phase drift scales linearly with fringe frequency and is monitored using registration marks (“notches”) that are laser-inscribed on one of the interferometer gratings (Englert et al. 2017b; Harlander et al. 2017; Marr et al. 2020) and imaged on the FPA.

In summary, the measured fringe phase shift, $\Phi _{\mathrm{A}}$, is related to the Doppler wind speed, v_A_, for each detector row (altitude), in the following way:

1 $$ \Phi _{\mathrm{A}} = \Phi_{\mathrm{A}0} + a^{*}(v_{s} + v_{\mathrm{A}}) + (f_{\mathrm{A}} - f_{\mathrm{C}})^{*}\Delta _{\mathrm{N}} + (\Phi _{\mathrm{C}} - \Phi _{\mathrm{C}0}) $$

with:

➢ $\Phi _{\mathrm{A}}$: measured phase versus pixel of the atmospheric airglow line observed for a particular tangent point altitude (FPA row) on the scene
➢ $\Phi $_A0_: fringe phase versus pixel of the atmospheric line for zero-wind (no Doppler shift due to wind or spacecraft velocity and zero interferometer drift)
➢ $a = (4\pi \sigma \Delta d / c)$, where $2 \Delta d$ is the path difference versus pixel, $\sigma $ is the signal wavenumber, $c$ is the speed of light
➢ $v_{\mathrm{s}}$: projection of the spacecraft velocity onto the MIGHTI line of sight direction
➢ $v_{\mathrm{A}}$: projection of the atmospheric wind velocity onto the MIGHTI line of sight direction
➢ $f_{\mathrm{A}}$: measured fringe frequency for the atmospheric line in units of radians per pixel
➢ $f_{\mathrm{C}}$: measured fringe frequency for the calibration line in units of radians per pixel
➢ $\Delta _{\mathrm{N}}$: measured shift of the grating registration marks in units of pixel
➢ $\Phi _{\mathrm{C}}$: measured fringe phase versus pixel of the calibration line
➢ $\Phi $_C0_: fringe phase versus pixel of the calibration line at zero interferometer drift

Solving equation (1) for the atmospheric and satellite Doppler velocity terms yields equation (2), below.

2 $$ a^{*}(v_{\mathrm{s}} + v_{\mathrm{A}}) = (\Phi _{\mathrm{A}} - \Phi _{\mathrm{C}}) - ([f_{\mathrm{A}}- f_{\mathrm{C}}]^{*}\Delta _{\mathrm{N}}) - (\Phi _{\mathrm{A}0} - \Phi _{\mathrm{C}0}) $$

There are three terms on the right side, each in parentheses, with the following physical interpretation: The first term is the difference between the atmospheric and calibration line fringe phases when the instrument is in the same thermal state for both. As discussed above, the thermal phase drift includes both interferometer drift, which is independent of the fringe frequency, and image shift, which is proportional to the fringe frequency (Englert et al. 2017a; Harlander et al. 2017; Marr et al. 2013). The second term in parentheses corrects the first term for the difference in phase drift between the atmospheric and calibration phases due to any shifting of the image. Note that this term depends on the spatial frequencies of the fringes and the shift of the notches, which is a measure of the image shift. The last term in equation (2) is the difference between the phases of the atmospheric line and calibration line when there is no atmospheric wind. This is the “zero-wind term,” which can be determined in a number of ways, as discussed in Sect. 3, below.

## Processing Approach for On-Orbit Wind Data

### Data Processing Flow

The two MIGHTI sensors produce images every 30 s (day) or 60 s (night). These images are passed in data-packets from the satellite to the data center at UC Berkeley, where they are recombined and saved as netCDF files. The raw fringe images are then processed and calibrated in several steps, as described below and, previously by Englert et al. (2017a) and Harding et al. (2017a). Once calibrated, the fringe phase versus optical path difference (OPD) is retrieved using Fourier Transform techniques. This phase is corrected, compared to a reference phase, and the resultant phase difference is compared to that from the zero-wind analysis, as shown in equation (2). The phase differences are inverted by altitude and, using the emission line wavelengths and instrument parameters, converted to get a horizontal wind profile, as projected onto the line-of-sight direction. Finally, measurements with orthogonal lines of sight from both sensors, MIGHTI-A and MIGHTI-B, are combined to derive altitude profiles of zonal and meridional winds, also called “cardinal” winds.

In the following, calibrated fringe phase data is also referenced as Level 1 (L1) data. Line of sight, vertical profiles of the horizontal wind are referred to as Level 2.1 (L2.1) data and cardinal winds (zonal and meridional winds) are also called Level 2.2 (L2.2) data.

### Stray Light Assessment

**Fig. 5:**
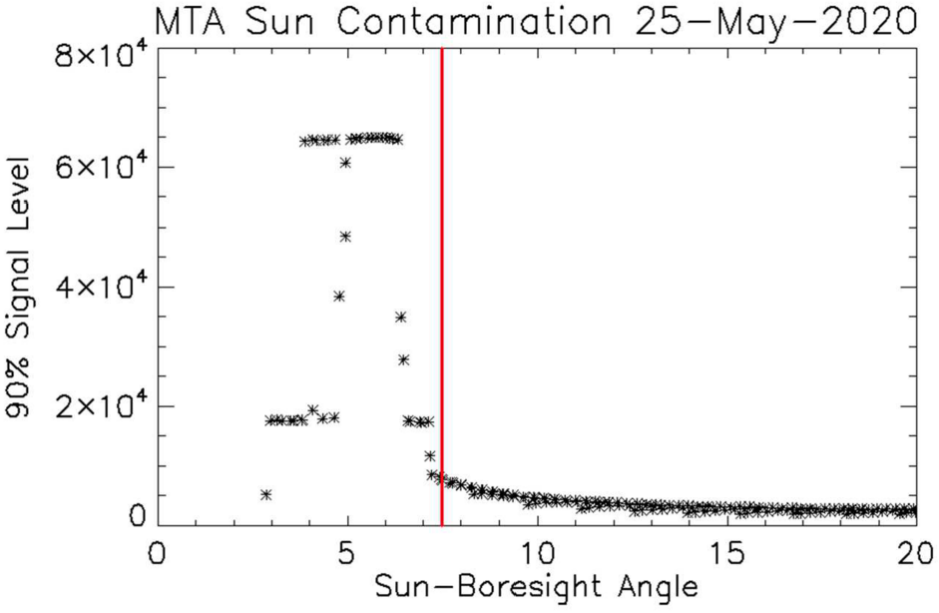
Contamination from the Sun as it approaches the field-of-view. Each point shows the highest signal level for 90% of the pixels in a single image. Saturation begins when the Sun-boresight angle is less than about 6 degrees. After July 26, 2021 the instrument shutter was closed for Sun-boresight angles of 7.5 degrees (vertical red line) or less to block the Sun from entering the instrument. Note that this solar contamination affects about 4% of the images, primarily near the terminators, on days when the beta angle of the ICON satellite is near 45 degrees

**Fig. 6:**
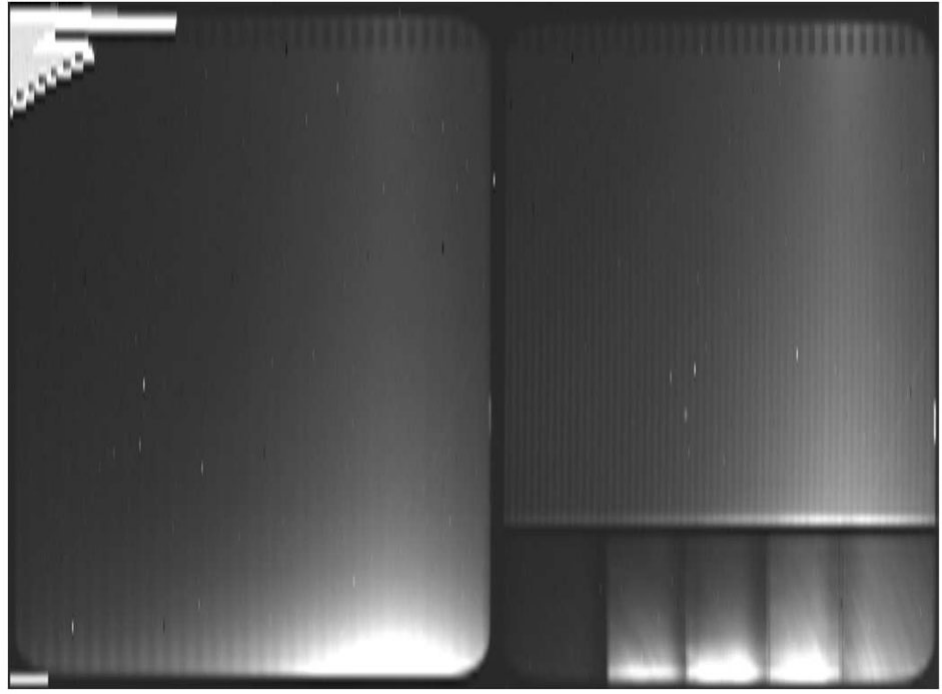
Sun entering the field of view from the upper left hand corner of the image. Sun to boresight angle changed from 8.1 degrees to 6.7 degrees during this 30-second exposure. The feature at the bottom right of the red-line fringe image is likely caused by internal reflections of direct sunlight entering the front baffle

The purpose of the baffle shown at the top of the left panel of Fig. 3 is to block light from outside the field of view of the instrument. The baffle, coupled with reducing the size of the input aperture during daytime operations, shields the instrument from bright cloud tops below the field of view, which are typically orders of magnitude brighter than the limb signals (Englert et al. 2017a). The vast majority of the MIGHTI data taken during normal operations show no evidence of scattered light from cloud tops or the hard Earth. However, there are a small number of exposures where the Sun or the moon come close or even enter the field of view. These observations provide a very stringent test of the instrument baffle. The Sun can get close to the field of view, when the field-of-view tangent point is near the terminator, typically for about 4 to 6 days during the summer when the satellite beta angle is near 45 degrees. Prior to version 5 (v05) of the wind data, the entire day’s worth of Sun-affected images were simply removed from the processing. However, it was found that on the days affected by the Sun, the dark images (see Sect. 3.3), typically taken near the terminators and therefore close in time to the Sun exposure, had anomalously high values, which were not representative of the dark signal for most of the day. These “contaminated” dark images could be avoided through the use of dark images and thermal drift calibrations which had been obtained prior to the Sun exposure and much of the data was able to be recovered in version 5. To avoid this issue after July 26, 2021, the instrument shutter is closed operationally when the Sun-boresight angle is less than 7.5 degrees. Figure 5 shows the signal level versus Sun-boresight angle for one of the affected days prior to Sun shuttering, indicating that the solar contamination becomes problematic for Sun-boresight angles of less than 7.5 degrees. Figure 6 shows a fringe image from the same day taken as the Sun is entering the field of view. During 2021, there were 1813 exposures excluded from the analysis pipeline due to proximity to the Sun, representing approximately 0.24% of the total exposures that year and approximately 4% of the images on days when the field of view moved close enough to the Sun to present an issue.

**Fig. 7:**
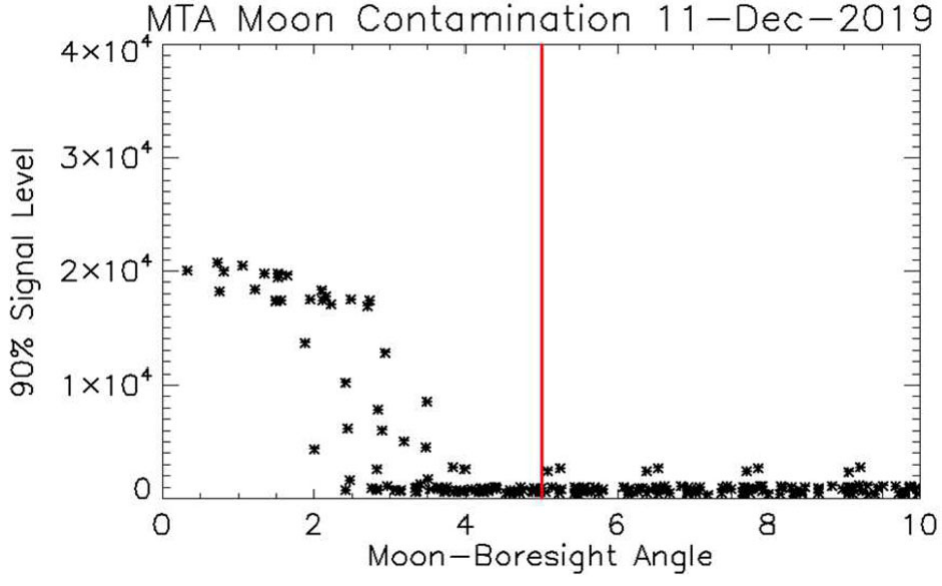
Similar to Fig. 5 but for moon contamination. Since the moon is much less bright, it can be closer to the boresight before the contamination becomes evident. Aperture shuttering has not been implemented for moon contamination, however, the Level 1 software removes images from the pipeline when the moon-boresight angle is less than 5 degrees (vertical red line)

The moon can also approach and even enter the field of view. Figure 7 shows the lunar contamination versus moon-boresight angle on an affected day. Since the moon is not nearly as bright as the Sun, we have not implemented shuttering for the moon, however, the data analysis pipeline rejects images when the moon-boresight angle is less than 5 degrees (indicated by the vertical red line in Fig. 7). Approximately 0.16% of the exposures during 2021 were removed from the pipeline due to the moon.

### Dark Current

**Fig. 8:**
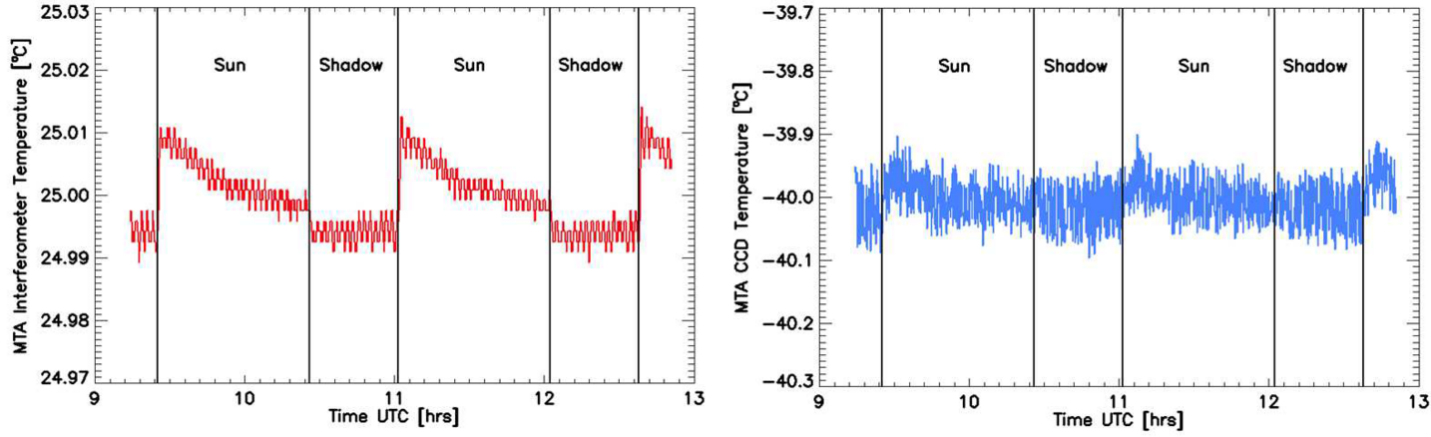
Measured interferometer temperature (left) and the CCD temperature (right) for MIGHTI-A covering two orbits on April 20, 2022. The vertical black lines indicate when the ICON satellite transitions from sunlit to shadow

Like every charged coupled device (CCD), MIGHTI’s focal plane arrays collect unwanted signal in each pixel over time as thermal energy in the silicon lattice produces spurious electron-hole pairs. This effect is commonly called “dark current” and typically increases over time as the CCD degrades from radiation exposure and other on-orbit environmental effects. To minimize dark current, the MIGHTI sensors employ CCDs that were designed to have a very low dark current and they are cooled to -40 °C to further minimize this effect (see Table 3 in Englert et al. 2017a). The right panel of Fig. 8 shows a typical example of the CCD temperature versus time, illustrating the temperature stability achieved on-orbit (<0.1 °C), facilitating dark current stability between calibration measurements.

**Fig. 9:**
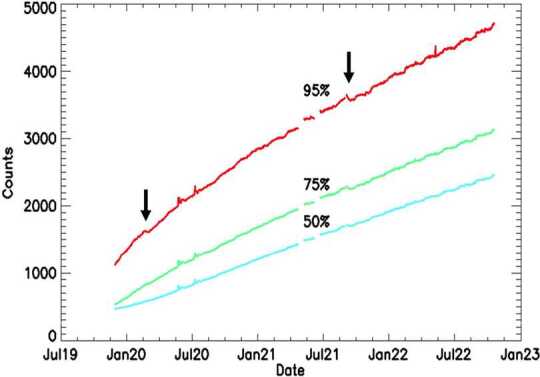
Increase in counts over time for MIGHTI-A 60-second dark exposures. The curves show the counts in ADU at the 50% (median), 75%, and 95% percentiles, for the typical MIGHTI binning of $2 \times 16$ pixels. MIGHTI-B behaves similarly. Small decreases can be seen in early 2020 and late 2021 (indicated by the arrows), when the instrument was turned off, causing the CCD to warm up to temperatures between -15 °C and -20 °C, for no longer than one day and three days, respectively, annealing some of the hot pixels

One result of the increasing on-orbit dark current is that dark images that are taken more than a week before the images being analyzed are no longer good representations of the dark signal in the image to be analyzed. In addition to the average increase in dark current for every pixel, which is commonly reported for CCDs in orbit, cosmic radiation passing through the CCD can generate additional signal within the pixels and typically causes sharp spikes in the images. The lattice damage caused by radiation can also create “hot pixels” that self-generate amplified dark current and appear as spikes in all subsequent images. These hot pixels are generally increasing in number and brightness throughout the mission. Collecting and averaging dark images close in time to the atmospheric and/or calibration images provides a means for reliably removing the dark signal and hot pixels from the atmospheric and calibration images. Figure 9 shows the “dark image” signal trends from November 2019 until October 2022 for the MIGHTI-A sensor. To quantify the average signal increase from spikes and hot pixels, we show three different trends in signal, using the raw ADU (analog to digital converter units) at the 50% (median), 75%, and 95% percentiles for the relevant CCD area. Small decreases can be seen in early 2020 and late 2021, when the instrument was turned off, causing the CCD to warm up to temperatures between -15 °C and -20 °C, for no longer than one day and three days, respectively, annealing some of the hot pixels.

An alternative reason for an increasing average dark current could be an increasing CCD temperature, which could be a consequence of a delaminating heat sink, which controls the CCD temperature. We considered this possibility, especially since the CCD temperature sensor is not mounted on the CCD, but on the heat sink directly behind it (see (Englert et al. 2017a, Fig. 13)). However, because we see a very similar effect for both MIGHTI-A and MIGHTI-B, and it appears almost linear in time (see Fig. 9), we assess this possibility to be unlikely.

**Fig. 10:**
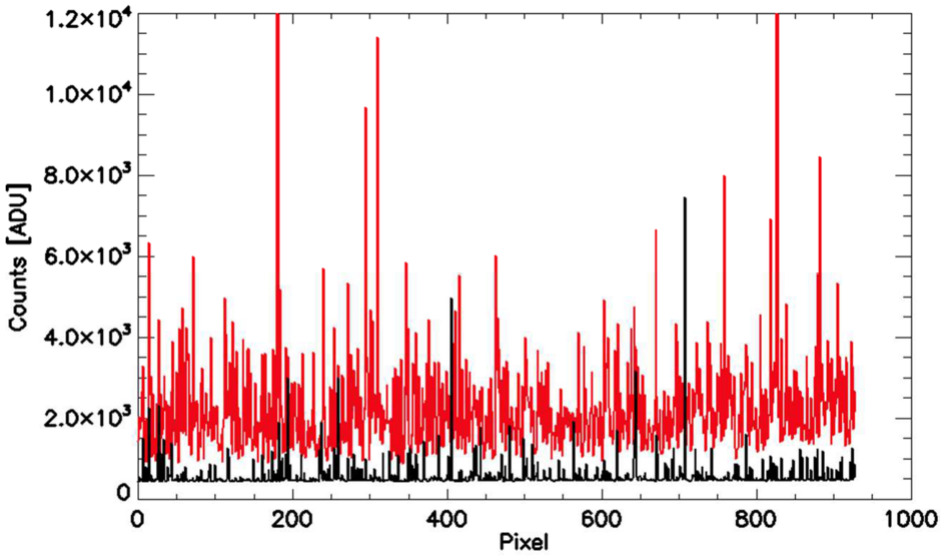
Representative dark signal for one row of the MIGHTI-A detector from December 2019 (black) and January 2022 (red) showing the increase in dark current and hot pixels. As a reference, pixel saturation occurs at a count level of 65,535 ADU

Figure 10 shows the dark signal for one row of the MIGHTI-A detector from December 2019 (black) and January 2022 (red), illustrating the marked increase in dark current and hot pixels.

### Spike Correction

MIGHTI’s on-orbit observations of the limb scene are subject to three physical processes that create spikes in the recorded image. First, stars will track through the image leaving bright streaks across the fringe patterns (see Fig. 4(B)). Second, cosmic rays passing through the CCD will create electron-hole pairs in the pixels and cause sharp, transient spikes in the data. Lastly, the damage caused by radiation will create hot pixels that self-generate signal and appear as persistent spikes in the image. Though these effects often can be removed through careful subtraction of dark images, this method is not always completely effective, e.g. when the hot pixel is relatively new or changing in intensity. Thus, it is important to employ an algorithm that removes residual spikes from observed images before further analysis, lest they cause errors in the retrieved fringe phase and wind speed.

**Fig. 11:**
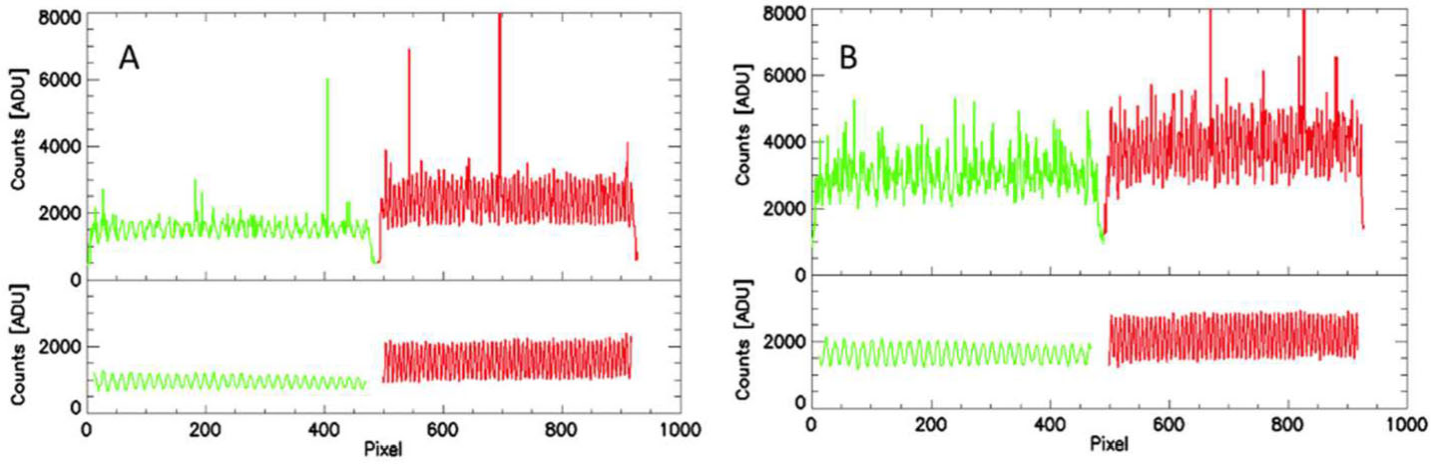
A row from a limb image before (top) and after dark subtraction, spike correction and flatfield correction (bottom). The left panels (**A**) are from a January 2020 observation while the right panels (**B**) are from an image taken in January 2022. The red and green traces correspond to the red and green oxygen emissions detected adjacently on the detector (see Fig. 4)

For the wind measurements, the MIGHTI spike correction routine analyzes images by horizontal row, along the fringes, for outliers and replaces them with values obtained from a spectrally filtered, smoothed version of the fringe. In practice, a row is extracted and Fourier transformed. The resulting spectrum is filtered to only retain power in narrow bands around the expected frequency components of an ideal fringe (e.g. near zero frequency, for the constant offset, and the expected frequencies of the fringes). A fringe with highly suppressed spikes is subsequently obtained by inverse Fourier transformation of the filtered spectrum. This smoothed fringe is subtracted from the original fringe to identify spike locations using a predetermined signal threshold. Signal values from the filtered fringe are used to replace the identified spikes in the original image. Note that away from the spikes the original values are not changed. Figure 11 (A) shows a row from a limb image acquired early in the mission before and after dark subtraction, spike and flat field correction. Figure 11 (B) shows a similar image from later in the mission.

### Flatfielding

Flatfielding for Spatial Heterodyne Spectroscopy instruments in general and Doppler Asymmetric Spatial heterodyne (DASH) instruments in particular has been discussed in detail by Englert and Harlander (2006) and Marr et al. (2012). In summary, flatfielding of a DASH instrument includes two elements: (1) the correction of pixel to pixel variability in the responsivity of the instrument to a spatially homogeneous source that is filling the entire field of view, and (2) the correction of any inhomogeneities of the fringe modulation efficiency (FME) across the imaging detector. For MIGHTI, both flatfield corrections, i.e. the “responsivity correction” and the “FME correction,” are accomplished using pre-launch data, as described by Englert et al. (2017a). In the following two sections, we show how MIGHTI on-orbit observations have been used to verify that the MIGHTI flatfields have not changed significantly during or after launch, and that the corrections applied to the flight data indeed achieve the desired effect.

#### Responsivity Correction

**Fig. 12:**
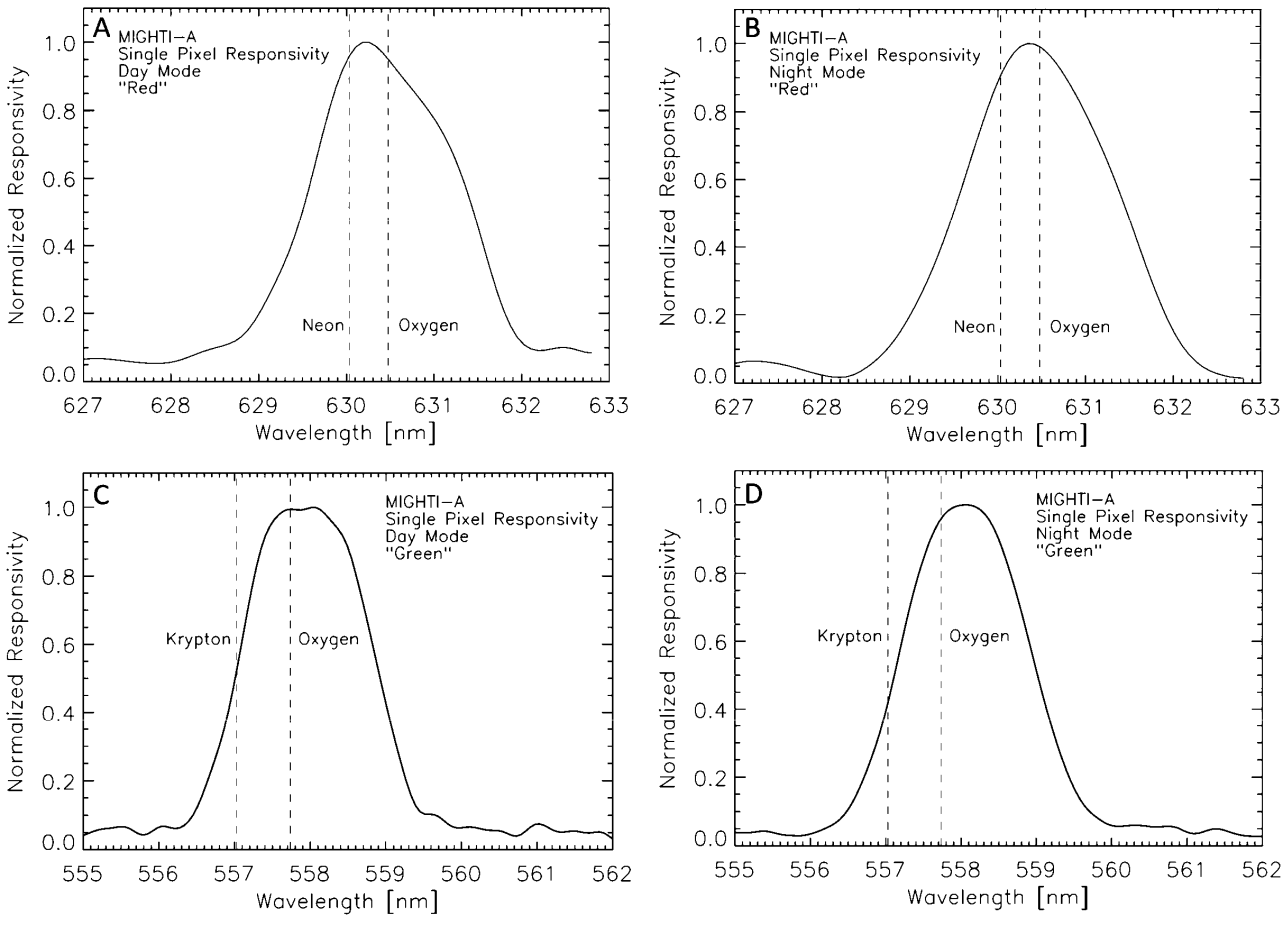
Normalized spectral responsivity of a representative binned pixel in the central part of the image, on the red (top plots) and the green (bottom plots) sides of the imaging detector of MIGHTI-A, together with the line positions of the respective calibration lamp emission lines (neon/red and krypton/green) and the oxygen airglow line positions. The left side plots are for day-mode operation and the right side plots are for night-mode

In order to achieve the responsivity correction, the spectral responsivity was measured in the laboratory for each pixel of the imaging detectors, using an integrating sphere that provided a spatially homogeneous signal source (Englert et al. 2017a). An example of the spectral responsivity for two particular pixels is shown in Fig. 12. The figure shows the spectral responsivity of a pixel in the central part of the image, on the red and the green side of the imaging detector of MIGHTI-A, together with the line positions of the respective calibration lamp (neon/red and krypton/green) and airglow lines (oxygen).

Figure 12 illustrates that the instruments’ spectral responsivity, which is predominantly determined by the spectral filters in front of the imaging detectors (see Fig. 3), was designed to be largest for the wavelengths of the oxygen airglow lines, while allowing the calibration lamps to still be within the passband, thus enabling efficient on-orbit calibration measurements.

MIGHTI has no on-orbit capability to measure a spatially homogeneous and spectrally narrow source, nor does it have any other special provisions to determine the responsivity at the airglow line wavelengths after launch. However, due to the significant spatial averaging of the observed scene that is resulting from the spacecraft motion during the integration times of 30 seconds and 60 seconds for day and night, respectively, the observed airglow signal can be approximated to be uniform in the horizontal direction. This allows the verification of the ground-based responsivity measurements for each individual horizontal row of the imaging detectors. Because neither the absolute brightness, nor the altitude dependence of the absolute scene brightness is known, only the relative responsivity, i.e. the pixel to pixel variations of the responsivity along a row, can be determined with on-orbit data. If this on-orbit, relative responsivity in the horizontal dimension is within the combined uncertainty of the ground-based and on-orbit measurements, it is reasonable to assume that the relative responsivity between the horizontal rows also has not changed between the ground-based calibration measurements and the on-orbit operation.

**Fig. 13:**
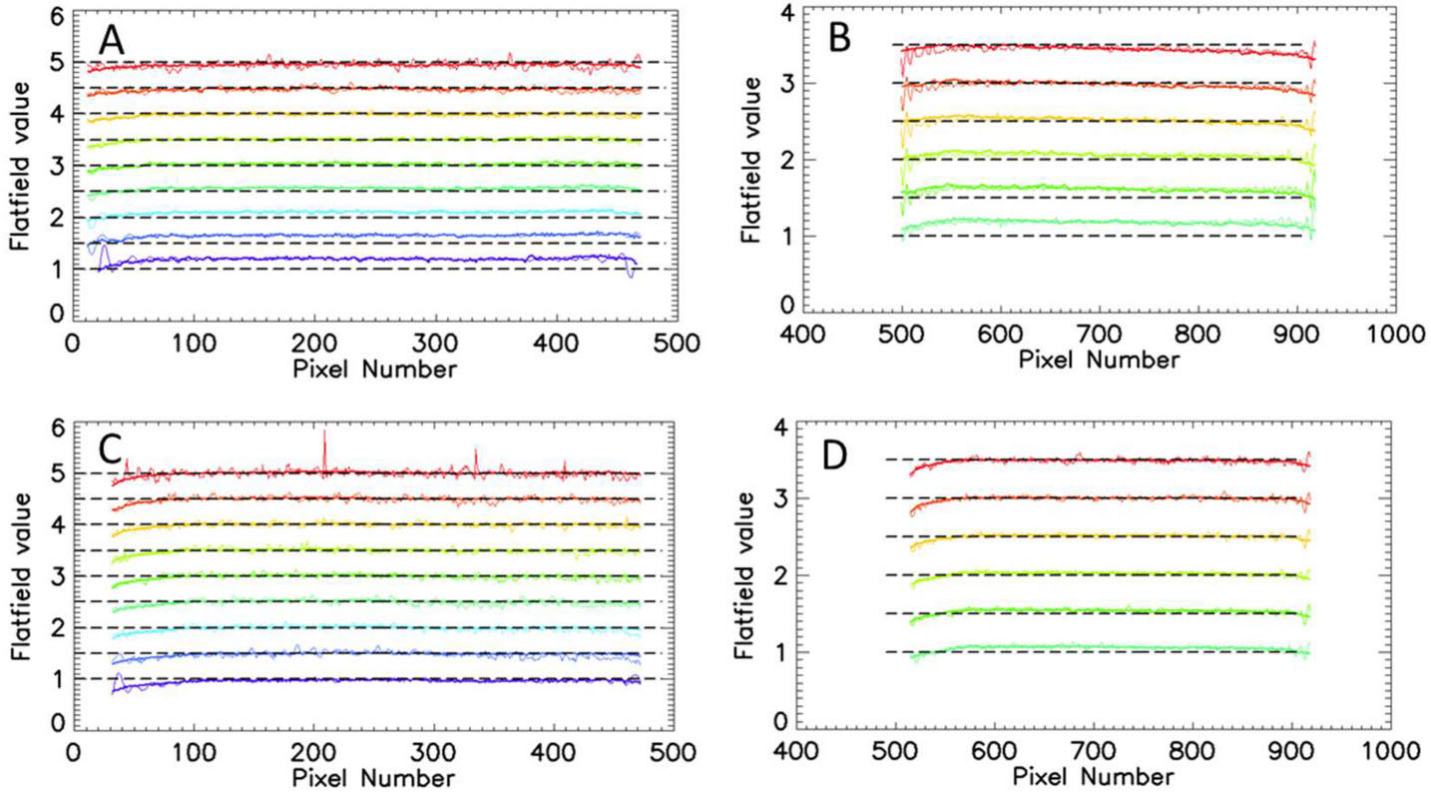
Panels A, B, C, and D show the relative responsivity for MIGHTI-A (red and green) in day-mode and MIGHTI-B (red and green) night-mode respectively, versus pixel number in several horizontal rows. The on-orbit data were obtained from the sum of approximately 700 limb images for day-mode and 270 images for night mode collected in April 2020. Each pair of traces shows the pre-launch relative ground-based responsivity (thick solid line) and the on-orbit derived relative on-orbit responsivity (thin solid line). The data are shown for equally spaced rows across the imaging detector (every 8th row). For clarity, the lowest plotted row is shown without a vertical offset while subsequent rows each have a vertical offset of 0.5 units from the previous row. The horizontal dashed line for each row indicates the ideal flat field value of one

To determine the relative responsivity along the horizontal rows of the imaging detectors, the unmodulated part of the atomic oxygen airglow signal in the interferogram was determined using the method described by Englert and Harlander (2006) and first applied to DASH interferograms by Marr et al. (2012). The value of the observed, unmodulated signal response along the rows of the imaging detector is then compared to the responsivity measured on the ground before launch. Representative results are shown in Fig. 13.

Figure 13 shows that the pixel-to-pixel variation of the ground-based measurements and the on-orbit data show very good agreement for each of the rows. This provides confidence that the responsivity of the instruments has not changed significantly during spacecraft storage and launch. Because the ground-based responsivity measurement has significantly higher precision than the on-orbit data, all MIGHTI on-orbit data are processed using the pre-launch responsivity data.

#### Fringe Modulation Efficiency Correction

The second part of the flatfielding is the correction of the non-uniform fringe modulation efficiency, which is determined by interferometer properties and the illumination of the interferometer (Englert and Harlander 2006). Similar to the responsivity correction, the FME, or fringe “visibility” correction, was also determined using ground-based calibration measurements. These FME measurements were obtained using neon and krypton emission line sources that are close in wavelength to the red and green oxygen airglow lines, uniformly illuminating the MIGHTI fields of view (Englert et al. 2017a). Note that the FME is also dependent on the line-width of the observed emission line, with narrower lines (lower temperature of the emitter) yielding higher visibility. Using the same data analysis method that was used for the pre-launch calibration (Englert et al. 2017a), on-orbit data of the atomic oxygen airglow emissions were used to investigate whether the pre-launch FME calibration data are still valid for the on-orbit observations.

The FME is the ratio of the modulated signal and the unmodulated signal and is therefore independent of the total signal level. Thus, under the assumption that the signal level is uniform for each horizontal detector row, and the linewidths of the observed lines are the same, one can determine the absolute, on-orbit FME for the entire illuminated detector area, rather than only in a relative sense, along one row, as is the case for the responsivity (see previous section).

**Fig. 14:**
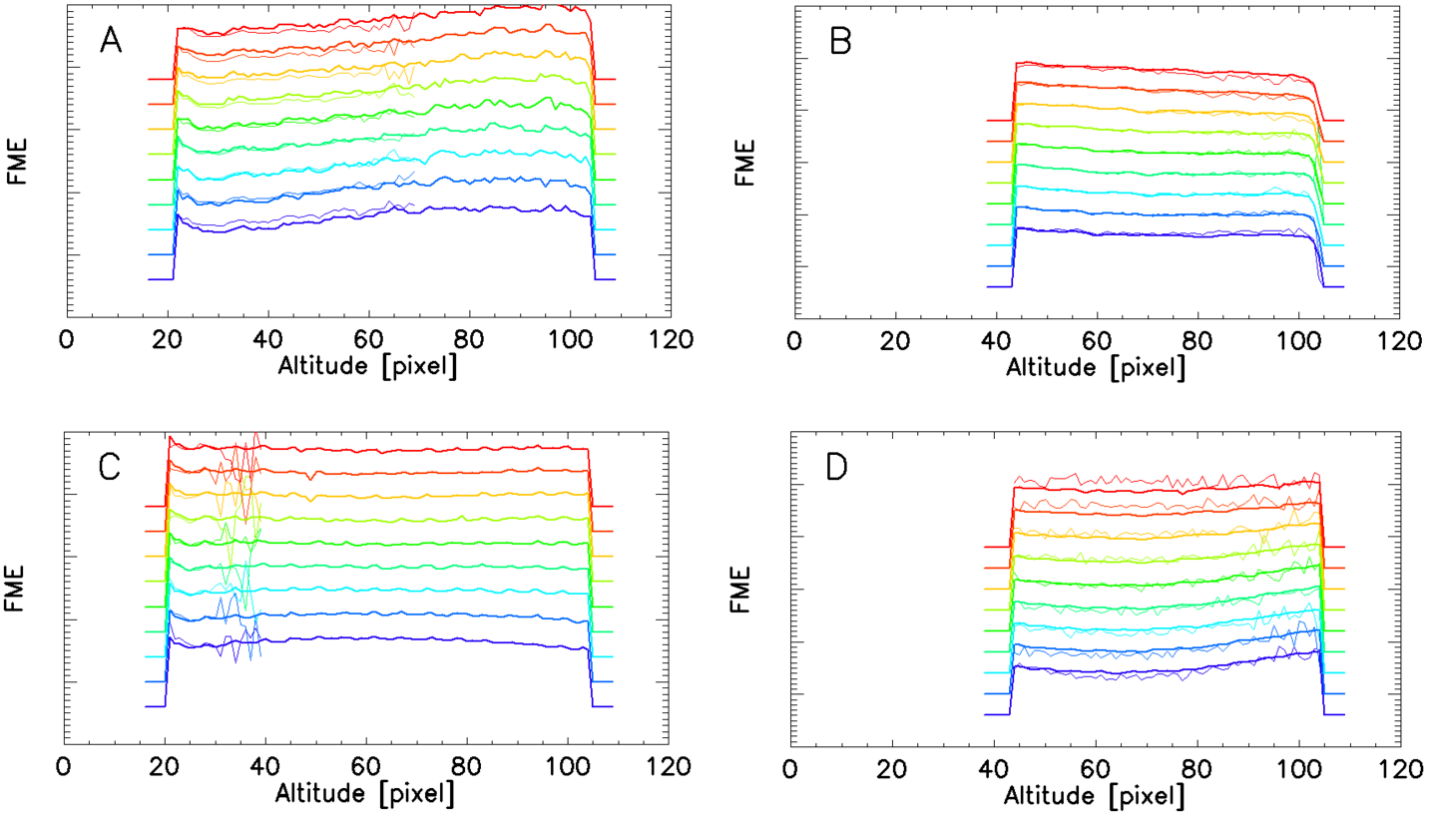
Panels A, B, C, and D show the fringe modulation efficiency for MIGHTI-A (green and red) in day-mode and MIGHTI-B (green and red) night-mode respectively. The on-orbit data were obtained from the sum of approximately 700 limb images for day-mode and 270 images for night mode collected in April 2020. Each pair of traces shows the pre-launch FME (thick line) obtained with laboratory calibration sources, and the on-orbit derived FME (thin line) obtained using the atmospheric oxygen emissions. The data are shown for equally spaced columns across the imaging detector (every 35th column) and an offset is applied to the data of the different rows for clarity. The short horizontal lines at the ends of each profile indicate zero visibility for that column. The ordinate (fringe modulation efficiency axis) is linear, but because it has a different offset for each color graph, we chose to not label the tick marks. Note that the on-orbit data in the green are limited to altitudes for which there is sufficient signal to measure the fringe modulation

Figure 14 shows the ground-based calibration data and the FME derived from the on-orbit oxygen airglow observations for representative vertical columns of the MIGHTI imaging detectors. At altitudes with strong airglow signals, the on-orbit data shows high signal to noise ratios and the pre-launch and post-launch results show reasonable agreement, especially when considering that the laboratory calibration sources and the atmospheric emission lines do not have identical line widths.

Some areas of the detectors are typically not illuminated by airglow signal, such as the altitudes above row #30 (∼150 km tangent altitude) at night for the green line. At these altitudes, the on-orbit derived FME cannot be determined with high precision using the atmospheric signal, but it is also not needed for the analysis of on-orbit data.

The good agreement shown in Fig. 14, in addition to the good agreement of the pre-launch and post-launch fringe patterns obtained from the calibration lamps (see Sect. 3.6) provides confidence that the FME of the instruments has not changed significantly during spacecraft storage and launch. Similar to the responsivity correction, because the ground-based FME measurement has higher precision than the on-orbit data, all MIGHTI on-orbit data are processed using the pre-launch FME data.

### Phase Distortion

Index of refraction variations and deviations from perfect flatness within the MIGHTI interferometers result in curved rather than straight fringes. Along a single row, these fringe distortions result in an unequal spacing of the fringes, i.e. a nonlinear relationship between fringe phase and optical path difference (OPD), represented as the pixel number along a row. These nonlinearities can be approximated as identical for fringes obtained from the on-board calibration lamps and the airglow signals because their wavelengths differ only by about 0.1%. During the prime mission it was found that this fringe distortion evolves slowly over time during normal operation and can experience discontinuities when the satellite enters safe mode and loses thermal control.

**Fig. 15:**
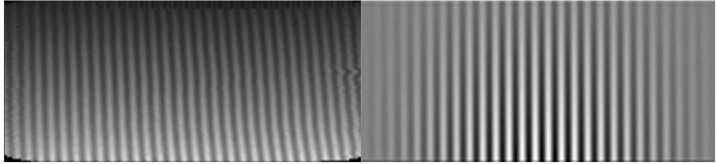
Green line calibration fringes obtained using the on-board krypton lamp before (left) and after (right) the phase distortion correction that straightens the fringes to facilitate smoothing (see also Harlander et al. 2019a, 2019b)

Following Eq. (2) for the determination of the atmospheric wind, the phase of the calibration fringes is subtracted from that of the atmospheric fringes. This means that the phase distortion, when applied to both the atmospheric and calibration phases, is removed in this subtraction, however, it should be noted that in order to minimize uncertainty introduced by the on-orbit calibration measurements, the calibration phases are smoothed prior to subtraction. To minimize possible systematic errors caused by this smoothing, the phase distortion over time is determined from the calibration lamp measurements themselves and then used to straighten the fringes (i.e. linearizing phase versus OPD as well as aligning adjacent rows) for both the calibration and atmospheric phases. During daily analysis after phase distortion correction (straightening), the calibration phases are fitted with a linear function, significantly improving the signal-to-noise ratio of the calibration phases. Figure 15 shows green-line calibration fringes pre- and post-straightening using the technique described by Harlander et al. (2019a, 2019b). Note that the straightened fringes have also been multiplied by a Hann function in the horizontal direction to minimize edge effects in the subsequent determination of the fringe phase.

**Fig. 16:**
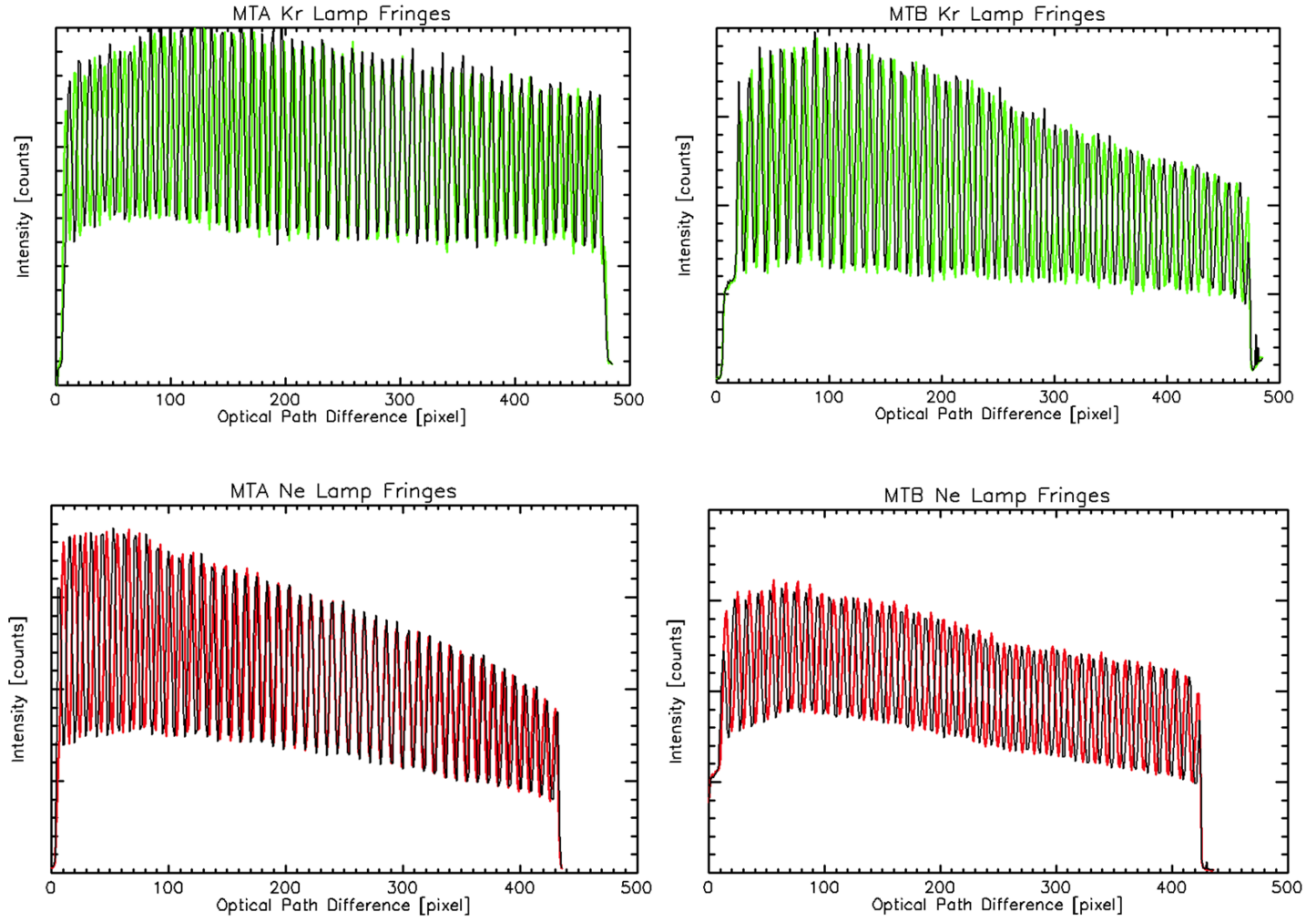
Pre- and post-launch fringes obtained with the on-board calibration lamps. Although the phase has changed, the brightness and contrast of the fringes has not, indicating no degradation of the interferometer performance for either MIGHTI-A or MIGHTI-B

The phase distortion is sensitive to small changes in either the index of refraction or in the dimensions of the optical elements, resulting in both thermal and secular changes of the phase response with time. Short term thermal changes have a periodicity associated with an orbit. These are discussed in Sect. 3.7 on thermal drift. Here we discuss the long term, secular changes and their incorporation into the phase distortion correction of the Level 1 data analysis. Figure 16 shows fringes obtained using the on-board calibration lamps, both pre-launch (green and red, acquired in June of 2016) and post-launch (black, acquired in October of 2019). Note that the fringe contrast and brightness are essentially identical, indicating that the quality of the fringes did not change during the more than three years between instrument calibration and launch. However, as anticipated, the pre- and post-launch phases of the fringes are slightly different due to a change in the phase distortion.

**Fig. 17:**
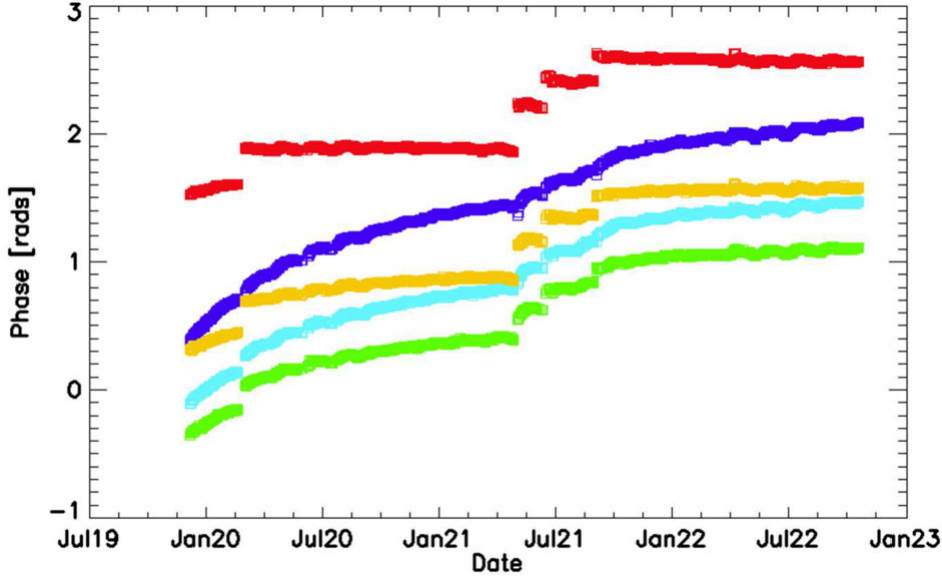
Plot of the time dependent phase for selected pixels, showing the evolution of the phase distortion versus time. The discontinuities occur when the interferometer thermal control was lost and reestablished. Phase observations using the calibration lamp signals and covering all relevant areas of the imaging detector are the basis for the phase distortion calibration used to straighten the fringes in the operational data analysis (see details in the text)

Figure 17 shows the phases of five pixels located near the center OPD of the MIGHTI-A red channel at different altitudes (FPA rows) obtained from measurements of the neon calibration lamp. Blue corresponds to low altitude and red to high altitude (rainbow order: blue, cyan, green, yellow, red). Note the steps in the phase plots, especially at the higher altitudes (red). These steps are coincident with losing and then re-establishing thermal control of the interferometer housing at times when the spacecraft went into safe mode due to on-orbit issues with the star trackers. After thermal control had been reestablished the interferometers settled into a new equilibrium state, resulting in the phase discontinuities. Between the discontinuities there is a slow drift of the phase that is most evident at low altitude (blue curve) but occurs at all altitudes. In practice, this trend is treated as an evolution of the phase distortion and is handled by fitting a quadratic polynomial between the discontinuities to the time dependent phase for each pixel separately. These fits are used to determine the time-dependent phase distortion calibration used to straighten the calibration fringes (linearize the phases) over the entire mission.

### Thermal Drift

As the spacecraft orbits the Earth in its low-inclination, low-Earth orbit and the solar illumination of the payload deck changes accordingly, the MIGHTI instrument elements experience significant changes in radiative heating and cooling. Even though the interferometers are thermally controlled to within less than 0.1 °C at the location of the temperature sensor inside the gold-plated interferometer enclosures (see Figs. 3 and 8), residual variations in the thermal environment of the interferometers can cause variations in the observed fringe phases throughout an orbit. Furthermore, thermal variations of the instrument’s optical bench, which is controlled to within ±2 K at around 20 °C, can cause changes in the relative positions and orientations of elements of the entrance optics, exit optics, and the detector, which, if not properly addressed, can be interpreted, incorrectly, as a phase change due to atmospheric wind. We examine both of these thermal effects and the associated corrections in the following two subsections.

#### Thermally Induced Phase Drift $\Phi _{\mathrm{C}}$

**Fig. 18:**
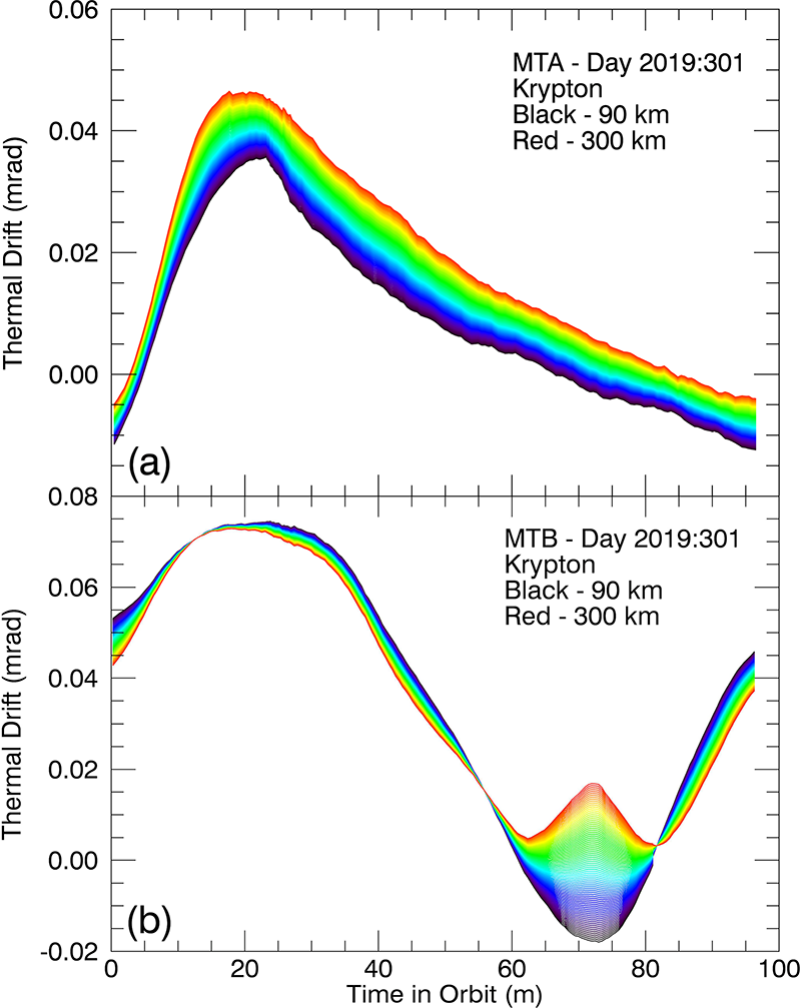
The thermal phase drift profile throughout an orbit for (**a**) MIGHTI-A and (**b**) MIGHTI-B sensors in normal operation from early in the mission. For these examples, the zero shift is chosen arbitrarily. Note that a phase shift of 1.8 (2.1) mrad is equivalent to a wind speed of 1 m/s for the red (green) oxygen airglow lines (see Table 1)

To track the phase drift throughout an orbit the neon and krypton calibration lamps are turned on during one orbit per 24 hour day. During this orbit, the “calibration orbit,” the signal from the calibration lamps is collected simultaneously with the atmospheric emission. In addition, in every calibration orbit, two images are taken that only contain calibration lamp signal. Finally, four dark images are taken during the calibration orbit. Using the phases from the calibration-lamp-only images as a reference, the on-orbit phase drift of the on-board calibration lamp signals is determined versus time, for all altitudes. This information allows the determination of the calibration phase, $\Phi _{\mathrm{C}}$ (see equation (1)), for all times during an orbit. Figure 18 shows the phase response to thermal variations during one orbit, for both the MIGHTI-A and MIGHTI-B sensors. Figure 18 illustrates that the shape of the response is significantly different for the two sensors due to their different orientations on the payload deck and the resulting differences in their illumination by the sun. The response versus altitude is also noticeably different between the two sensors.

#### Image Shift Correction

As mentioned above, the change in the recorded fringe phases of the calibration lamps can be attributed to two fundamentally different effects: Thermal changes within the interferometer and thermally induced movement of the fringe image on the CCD. As discussed in Sect. 2, the second effect does not result in the same phase change of the calibration signal and the atmospheric signal, so that a correction term has to be included, which depends on the fringe frequency difference and the movement of the image (see equation (1)).

The image movement on the CCD, or image drift, can be determined using the image of a regular (comb) pattern of registration marks, or notches, that are laser-inscribed on one of each interferometer’s gratings (Harlander et al. 2017; Englert et al. 2017a,b). The notch positions can be retrieved for every image for which the calibration lamps are on. Images with only atmospheric signal do not provide enough illumination of the parts of the gratings that contain the notches.

##### Determining the Notch-Pattern Position

The position of the notch pattern is determined through a least-squares fitting routine which assumes a priori knowledge of the width, depth, and rounded shape of the individual notches in the pattern, similar to the one described by Marr et al. (2020). By using the shape of the calibration line fringe pattern, as determined from the fringe image in a row just below the notches, this method reconstructs an oversampled fit to the row of notches with a 4-parameter fitting routine. The four parameters are the notch pattern position, the fringe amplitude inside the notches, the fringe amplitude between the notches and a scalar for a constant offset. From the value of the notch pattern position of the individual image, and the corresponding one from the calibration-only image, we derive $\Delta _{\mathrm{N}}$ (see Eq. (1)). Using this technique, the horizontal notch pattern position can be determined, for each individual image, with a precision of about +/- 20 (50) milli-pixels for MIGHTI-A (MIGHTI-B), which corresponds to an equivalent wind of +/- 3.4 (7.8) m/s for the green-line and +/- 8.5 (20) m/s for the red-line. The difference in uncertainty is mainly due to the difference in the illumination of the notches by the calibration lamps.

The examination of the on-orbit data revealed that the temporal dependence of the term $\Delta _{\mathrm{N}}$ can be separated into components with three distinct time scales. First, there is a component that is highly periodic, on the time scale of one orbit, as the satellite goes in and out of the sunlit part of the orbit. Second, there is a component that is also periodic and dependent on the precession cycle, changing with the orbit’s beta angle and the resulting changes in solar illumination. Third, there is a long term drift term, which is likely associated with the mechanical “settling” of the instrument, driven by stresses within the mechanical structure and possibly even aging of the materials.

While all three components could theoretically be tracked using the notch-pattern position, the analysis of the data showed that better precision of the correction can be achieved using three separate approaches for the correction, which are described in the following. In short, the intra-orbit variation is quantified and corrected using the notch position, as described by Englert et al. (2017a) and Marr et al. (2020). The precession cycle effect is quantified and corrected using the fact that the image shift has a different effect on the red-line and green-line winds, and for altitudes at which both emission lines can be used to measure the winds, the retrieved wind values have to be the same. Finally, the long-term image drift is similar to the effect of the long-term drift of the “zero-wind” phase, the phase expected for a simply co-rotating atmosphere (no wind). Therefore, the correction for this effect is included in the correction of the zero-wind phase, which is described in Sect. 3.11 below.

##### Intra-Orbit Image Shift Correction

**Fig. 19:**
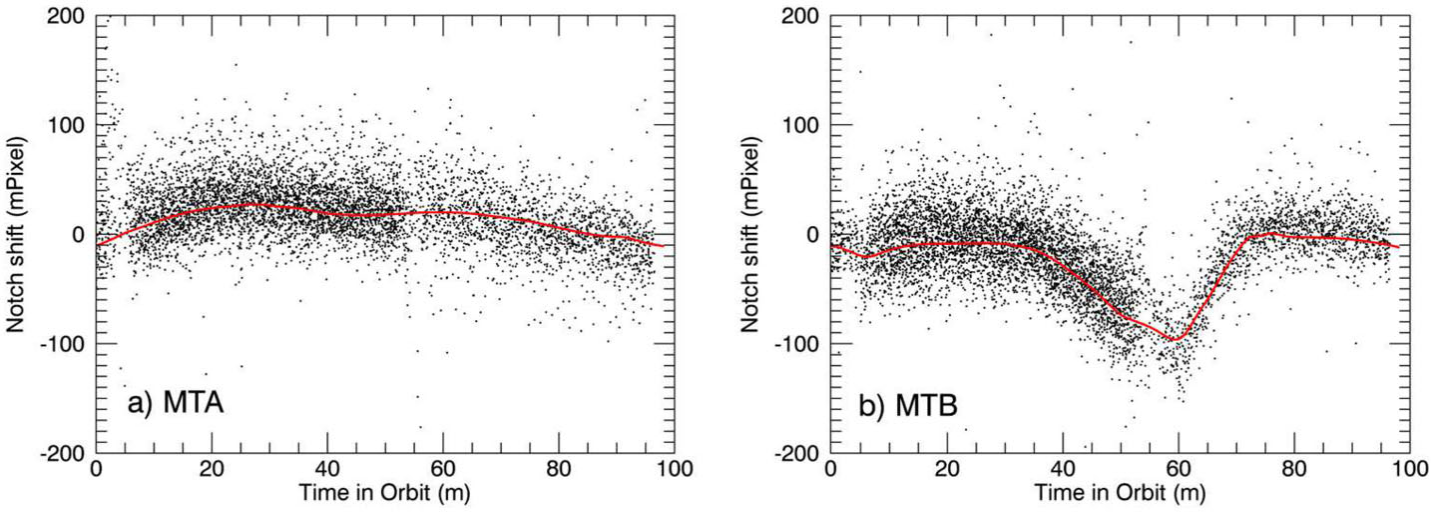
(**a**) The black dots are the results of the time-dependent notch position fits for every image in fifty calibration orbits of MIGHTI-A. This includes data from both neon/red during the day and krypton/green at night. The red trace is the high-precision shape of the intra-orbit image shift determined from these data. In daily wind analysis this shape is simply offset to best match the notch positions determined from the calibration orbit that day. (**b**) Similar to (**a**), but for MIGHTI-B. Note that a notch shift of 2.48 (6.68) mPixel is equivalent to a wind speed of 1 m/s for the red (green) oxygen airglow lines (see Table 1)

Using the images from the once-daily calibration orbits, the short-term, intra-orbit image position change can be monitored and corrected. It was seen that even between day mode (Aperture A2 about 15% open, see Fig. 3) and night mode (A2 100% open), and between neon and krypton calibration lamp data, the shape of the intra-orbit notch shift remained nearly consistent. By combining notch positions from many days worth of calibration orbits, representative shapes for the notch position change throughout an orbit were determined with high precision. Figure 19 shows these orbital image shifts (red) for MIGHTI-A and MIGHTI-B overlaid on a sample set of the data from fifty days of the mission.

The analysis of calibration-orbit data throughout the mission shows that the shapes shown in Fig. 19 continue to be a good representation of the intra-orbit image drift for the entire mission to this date. Fitting the high-precision shapes to the daily calibration orbit data significantly reduces the uncertainty of the value for $\Delta _{\mathrm{N}}$, required in equation (1) for the correction of the intra-orbit image-shift effect.

##### Precession Cycle Image Shift Correction

**Fig. 20:**
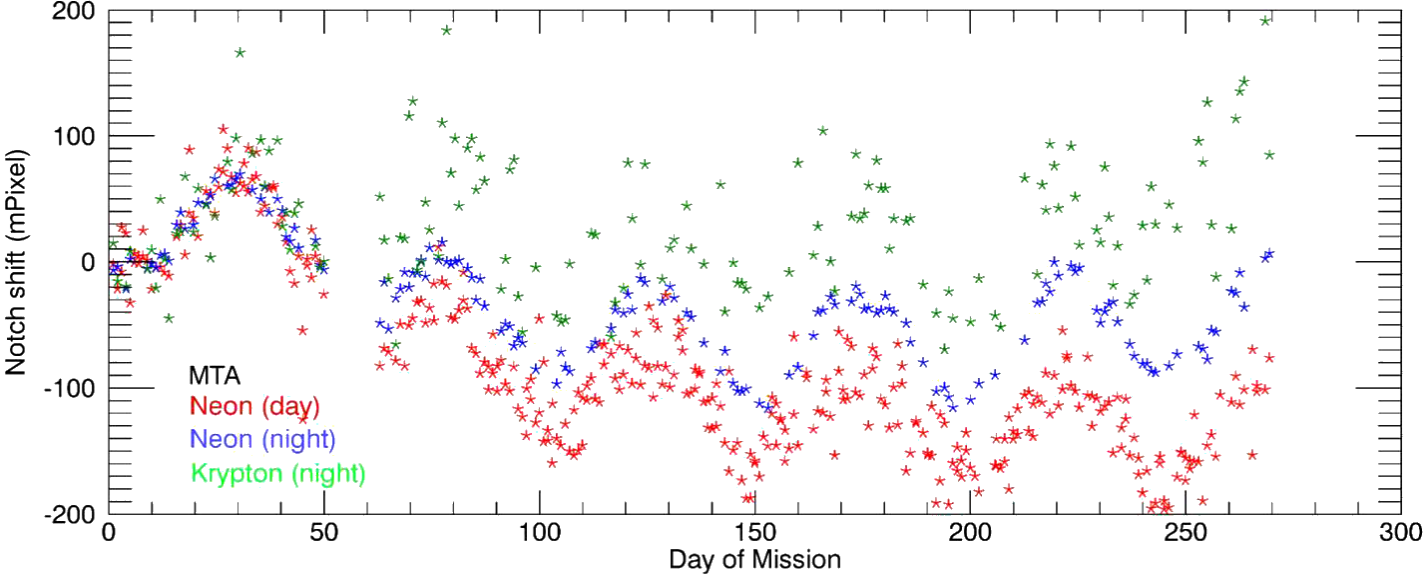
Measured image position shift for nearly one year of daily MIGHTI-A calibration lamp images (2020). The red (blue) stars are the notch shifts determined from the neon day (night) calibration-lamp-only images and the green stars are from the krypton lamp observations at night. Krypton day-mode results are not shown, due to the low signal level for this lamp-aperture combination. The reference notch position was arbitrarily chosen as the average position of the first ten days of 2020. Diverging image positions for day and night modes, and between red and green images are apparent as the year progresses

Figure 20 shows an analysis of daily averaged image shift data, which reveals a significant 48 day oscillation with an amplitude of about 50 milli-pixels, indicating a clear precession cycle dependence. In addition, the data shows diverging image positions for day and night modes, and between neon and krypton images as the year progresses. Note that the day-to-day variability shown in Fig. 20 is dominated by the rather low precision in determining the notch positions, as discussed earlier. To achieve a high precision correction for this effect, together with consistency between the green- and red-line wind data, the differing effect of the image shift on the green- and red-line winds is exploited. As indicated in equation (1), because the fringe frequencies $f_{\mathrm{A}}$ and $f_{\mathrm{C}}$ of the green and red, atmospheric and calibration lamp fringes are different, a different image shift $\Delta _{\mathrm{N}}$ has a different effect on the retrieved wind and only one $\Delta _{\mathrm{N}}$ will result in the same wind result for wind retrievals of the different color emission lines. Thus, at altitudes and local times for which both green and red line retrievals are possible, a unique solution for $\Delta _{\mathrm{N}}$ can be found.

For version 5 (v05) of the wind data product, a precession cycle image shift correction was determined using data over the altitude range of 167–185 km, which is a region where both emissions are suitably bright, yielding high precision wind results for both colors. In addition, the local time interval between 0900 and 1500 is chosen to avoid potential artifacts near the solar terminator. To determine the desired precession-cycle-dependent image shift, we first perform a linearized approximation of the inversion described by Harding et al. (2017a). This simplified inversion is sufficiently accurate for this purpose, even though for the red-line, the selected altitudes are mostly below the peak altitude of the airglow emission. The L1 fringe phases (after being subject to spacecraft velocity subtraction and the approximate inversion) are analyzed to find the daily-median green-red difference. Because the data shown in Fig. 20 does not show a significant image shift structure with higher frequency than the precession cycle, a 13-day rolling median filter is applied to the result, to increase the precision of the correction, without significantly reducing the amplitude of the variation. In addition, a 48-day rolling median is subtracted, to isolate the precession cycle contribution of the image shift from the long term drift. As mentioned above, the long term drifts shown in Fig. 20 are corrected by the zero-wind correction, discussed in Sect. 3.11.

**Fig. 21:**
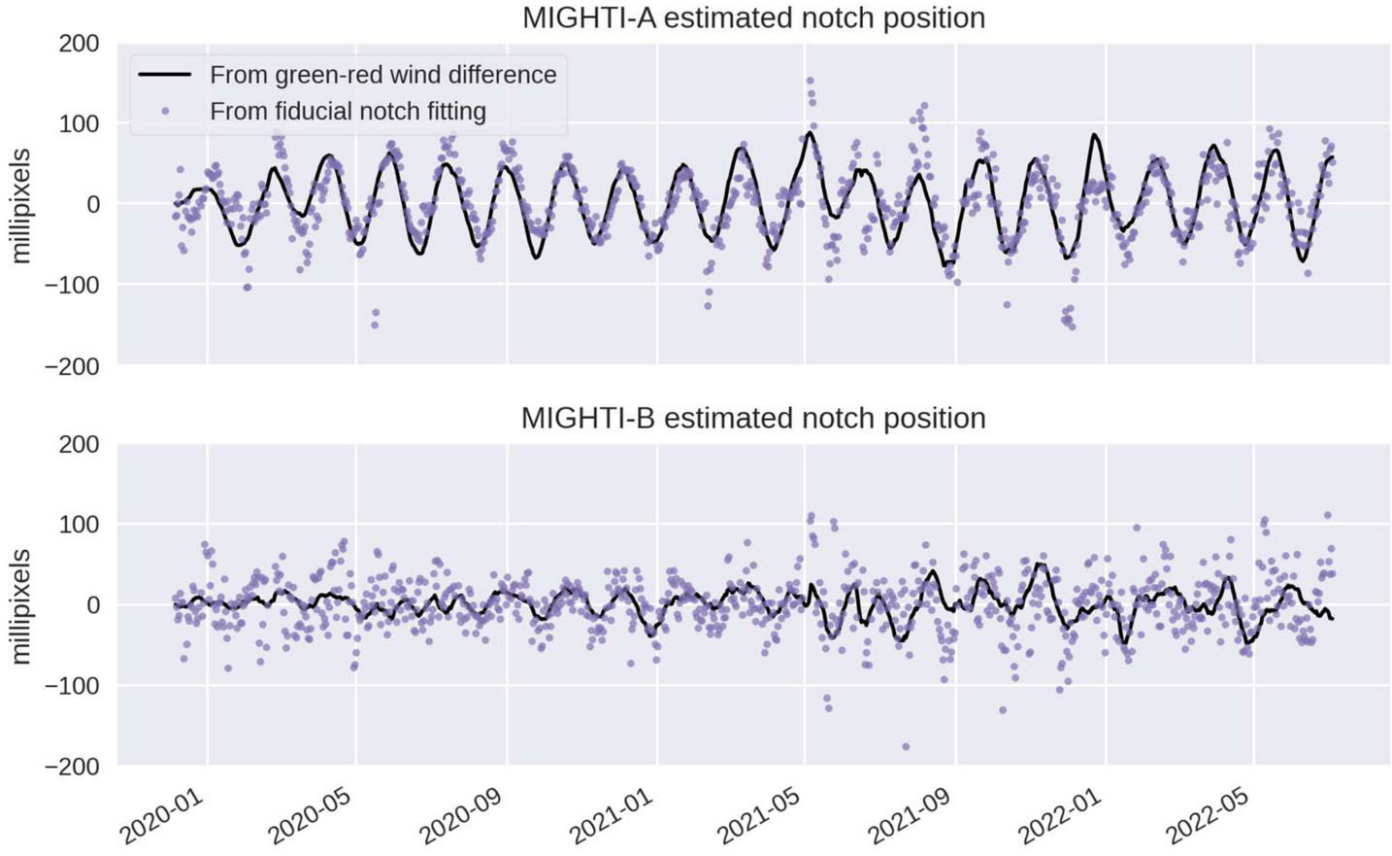
Two ways of characterizing image shift on the CCD are shown. The black line is based on the condition of equal winds for the red- and green-line retrievals (used operationally for v05 data), after the removal of the longer term drifts via subtraction of a 48 day mean. The purple dots are from first-principles fits of the fiducial notch pattern, similar to Fig. 20. A 48-day rolling-window median has been removed from these latter data for consistency. The data shows clear similarity between the image shifts oscillations with the frequency of the precession cycle

The resulting image shift patterns used for v05 wind data are shown as the black lines in Fig. 21. First-principles fits of the day-mode fiducial notch pattern (similar to the red symbols in Fig. 20), are shown as purple dots. The similarities confirm that the result derived from the green-red difference is consistent with the notch positions, at least in terms of the intra-precession-cycle trends. The similarities of the features on the timescale of the precession cycle, rather than longer term trends, show that the above described approach accurately represents this contribution to the image shift.

To validate the image shift correction, a comparison between winds retrieved from the red- and green-lines (Level 2 data) is presented in Sect. 6.1. In short, the results show that the red-green differences are centered near zero, and the discrepancies can be accounted for by known, specified uncertainties. This result shows that this image shift correction approach was successful at suppressing precession cycle artifacts associated with mechanical drifts. Future work on the notch fitting algorithm will determine whether, in the future, the notch positions could be determined with sufficiently high precision for all local times, instrument mode and color combinations, to directly calibrate the data, which would allow the red and green data products to be entirely independent, rather than cross-calibrating them to determine the precession-cycle trends.

### Stellar Calibration and Instrument Pointing

Accurate knowledge of the instrument pointing is needed for geo-referencing the data, but the most critical need for pointing knowledge is the correction for spacecraft velocity. The wind estimate is required to have a precision on the order of 10 m/s, yet the tiny Doppler shifts from wind variations are superimposed on top of large Doppler shifts from spacecraft motion (approximately 7000 m/s). The requirement for ICON pointing knowledge is 0.01° (equivalent to about 1 m/s uncertainty in speed). ICON’s attitude control system was designed to meet this requirement, and post-launch commissioning verified its performance. The on-board star trackers constantly deliver the quaternion which defines the orientation of the spacecraft body frame in inertial coordinates to within this precision.

The missing piece of information is the orientation of MIGHTI’s field of view (FoV) in the spacecraft body frame. This was determined using a special maneuver that points the MIGHTI FoV to a dense region of stars (a region containing the Pleiades star cluster) and takes a high-resolution image (no binning of the CCD pixels). A ground-based, pre-launch calibration quantified the look direction of all MIGHTI pixels relative to an arbitrarily-defined “boresight” pixel, using a high-order polynomial to capture deviations from a linear plate scale. Six bright stars were manually located in the image, and the polynomial was used to convert the observed star pixels to angles relative to the boresight (i.e., the unit vectors of star locations in the “MIGHTI frame”). Using this information, the quaternion can be determined that, when applied to the observed positions of stars, minimizes the squared difference with the known positions of stars in the spacecraft frame (as determined by a star catalog and the star-tracker quaternion).

Two stellar calibration maneuvers were performed 24 days apart (29 Oct 2019 and 22 Nov 2019). The pointing was determined independently using these two datasets, and the difference (when converted to an effective spacecraft velocity projection) was less than 0.6 m/s. While this test does not rule out any systematic errors in the maneuver or the algorithms that are common to both analyses, it provides confidence in the results.

**Fig. 22:**
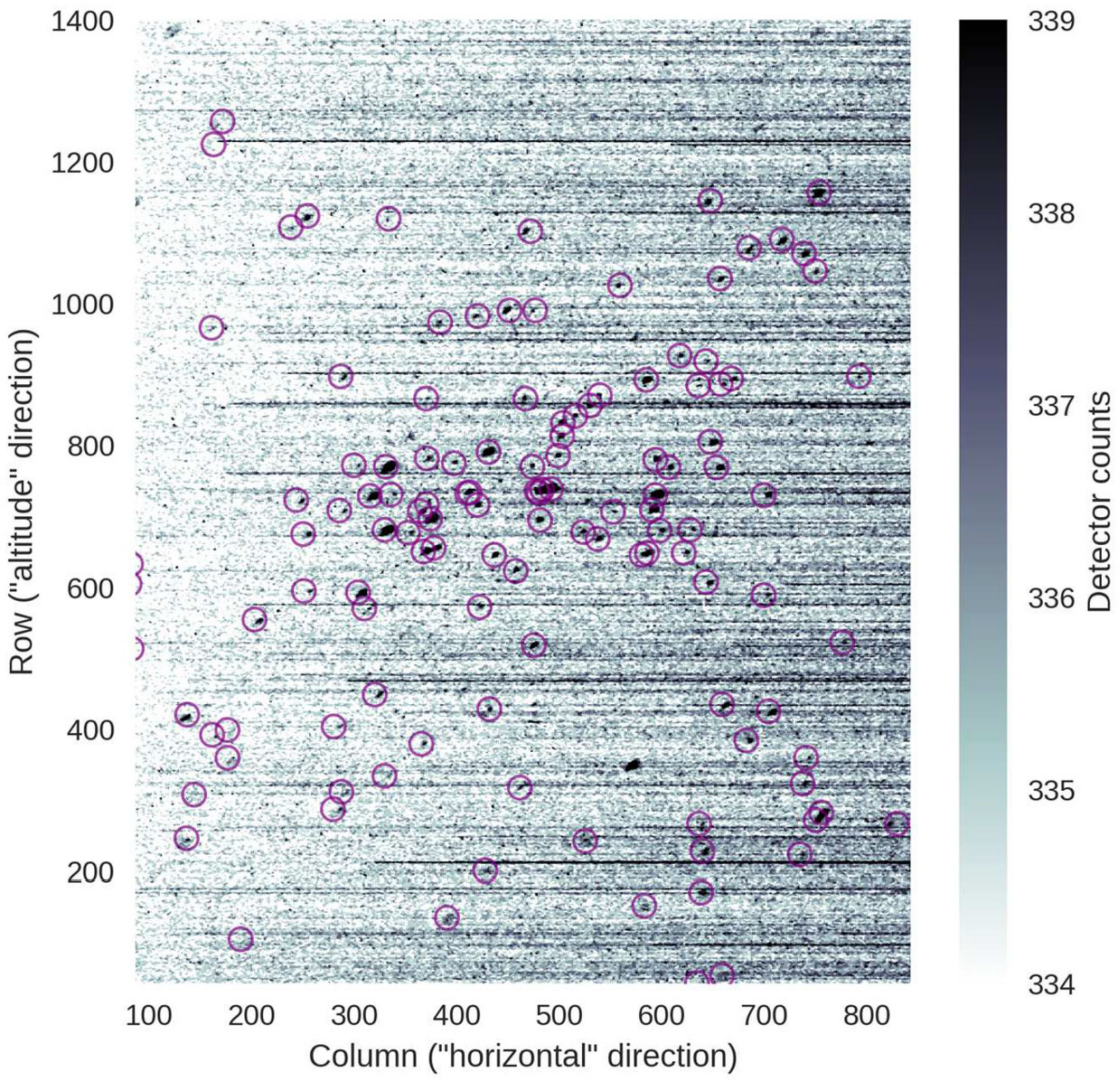
A stellar calibration image (gray-scale) compared to expected star locations (purple circles), calculated from the spacecraft-to-inertial-frame quaternion as reported by the star tracker and the solved-for MIGHTI-frame-to-spacecraft-frame quaternion. The image has been processed by taking the median of 3 successive images (on a per-pixel basis) and then applying a 3x3-pixel median filter, to reduce noise. Each image had an integration time of 30 seconds. Shown is the green channel, and only the rows and columns actually used for science data. Horizontal streaks are artifacts associated with hot pixels

Figure 22 shows the result of an end-to-end verification of the pointing determination. The observed image is shown, along with expected locations of stars, as determined by the Smithsonian Astrophysical Observatory Star Catalog (SAOSC 2001), the star-tracker’s quaternion, and the instrument quaternion we determined above. Figure 22 shows that, in addition to the six stars used to determine the pointing, the location of other stars across the image are accurately predicted without revealing any obvious additional distortions.

In normal science mode, the spacecraft is commanded to keep the tangent points of the MIGHTI-A and -B image bottoms at about 90 km. The actual attitude, as determined by the on-board star tracker data is used in all subsequent processing (e.g., geo-referencing and spacecraft velocity correction).

### Sufficient Signal Verification

Even though many steps are taken to isolate the modulated signal from the dark current, spikes, and constant offset, there are times that the signal is not bright enough to yield a reliable wind retrieval, especially at the top of the airglow layers. The simplest time to check for sufficient signal is during the search for the peak of the emission line in frequency space, after the Fourier transform of the interferogram. If, in the power spectrum, the magnitude of the emission line peak is not at least ten times larger than the standard deviation of the noise away from the peak, then the signal is deemed too low to be analyzed. Further signal-level checks are performed throughout the subsequent processing. For example, if not enough data is available at a particular altitude over the precession cycle, the zero-wind phase determination will fail and no data will be reported at that altitude (e.g., the nighttime red line wind below ∼200 km). Also, a row of the interferogram may have sufficient signal at Level 1, but might yield negative signal after the inversion (e.g., between the two brightness peaks of the green-line emission profile near the terminator). These are identified during the inversion and set to zero, as negative signals are not physical.

### Low-Signal Phase Shift

**Fig. 23:**
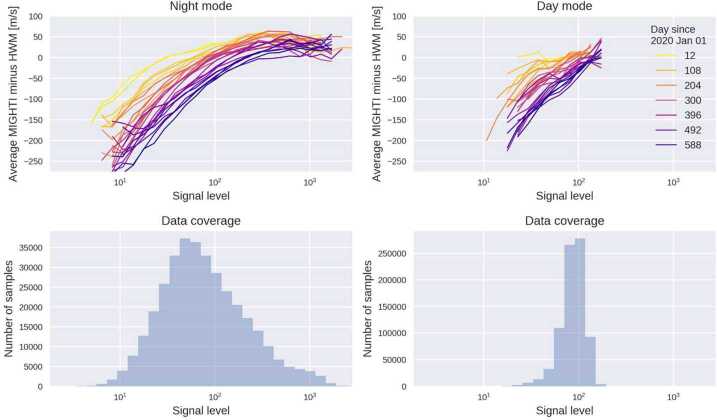
The difference between MIGHTI wind estimates and HWM14, binned as a function of signal level. Data between Jan 2020 and Oct 2021 are analyzed, with each 48-day precession cycle analyzed independently, resulting in the colored lines (top panel). The histogram of signal level is shown in the bottom panels. The signal level is defined as the DC value of the interferogram during the day, and 13.3 times that value at night (to account for the increased sensitivity). A clear negative systematic error is seen at low signal levels. Data from the red-line channel at ∼244 km altitude in MIGHTI-A are shown. Green-line results are qualitatively similar, except the histograms are shifted to the right (i.e., there is less of an effect on most green-line wind data). MIGHTI-B results are similar

As limb observations were obtained over a wide range of limb brightnesses, it became apparent that there was an unexpected, systematic shift in fringe phase depending on the signal strength on the detector, which was determined to be characteristic of the instrument rather than geophysical. This shift was strongest for the dimmest signals and tended toward zero shift at the brightest signals. The top panels in Fig. 23 show the measured red-line wind at 244 km binned as a function of signal level. The binning is done separately for each 48-day precession cycle, each of which is shown as a colored line. The lower panels show the histogram of samples that were used to generate the top panels. Note that the signal level is defined to account for the different instrument sensitivities in day mode and night mode – the night-time DC values (unmodulated signal on the interferogram) have been multiplied by 13.3 and the day-time by 1.0. The multiple curves are for different time periods, coded by color, with darker colors representing times later in the mission. These curves indicate that the phase shift is increasing with time. Note that establishing zero-wind as discussed in Sect. 3.11 “corrects” all curves near the peaks of the frequency distributions so the wind biases will effectively be removed near the peaks of the distributions shown on the lower panels (i.e. the most common signal levels).

**Fig. 24:**
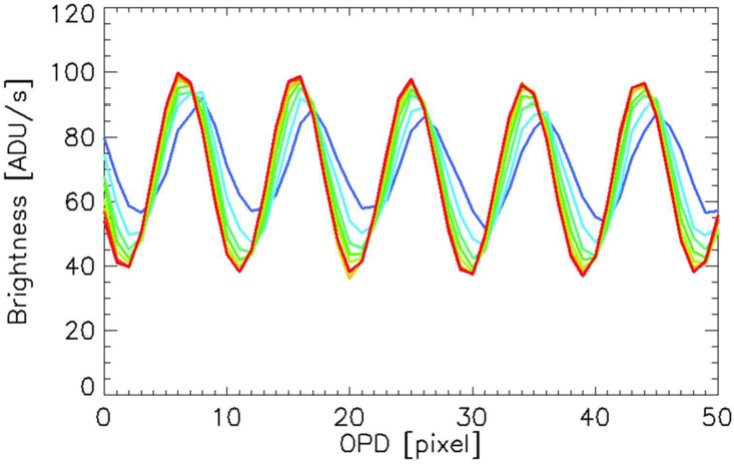
Sample fringes indicating fringe phase shift depending on the total signal detected in a given CCD row. The measurements were made on January 2, 2022 using only calibration lamp signal. The colors represent different exposure times (total signals) bracketed by red at 240 seconds and blue at 2 seconds. For clarity, only about one eighth of the row is plotted

To further investigate this effect, special observations of the on-board calibration lamps have been made with eight different exposure times to produce different total signal levels at the CCD. Figure 24 is a plot of the MIGHTI-A neon fringes from these calibration-lamp-only measurements obtained on January 2, 2022. The different colors correspond to the exposure time of the observation, bracketed by the red curve at 240 seconds and blue curve at 2 seconds. The vertical axis is in ADU per second which normalizes each of the curves to exposure time. Each of the curves has approximately the same mean value near 70 ADU/sec indicating the expected brightness scaling with exposure time, however, as the exposures become shorter (less total signal) there is clearly a progressively larger phase shift and a decrease in fringe amplitude. Since these measurements were obtained with the calibration lamps, the phase shift and amplitude reduction is purely an instrumental effect. The cause is under investigation but these results indicate the shift is consistent with a linear “smearing” of the fringes, perhaps in the readout process along the rows, which affects the faint, short exposures (blue curve) more than the bright, longer exposures (red).

Although the root cause is still under investigation we have implemented a correction for this effect based on both the limb and calibration lamp measurements. The correction is slightly different for MIGHTI-A, MIGHTI-B, day and night but in all cases is determined from measured phase shifts with different signal levels. The correction curves asymptotically approach zero at the brightest signal levels. To account for the time dependence indicated in Fig. 23, the correction increases linearly in time from the start of the mission with indications of leveling off during the third year on orbit. The uncertainty associated with the correction is conservatively estimated to be 40% of the correction and has been included in the overall wind uncertainty discussed in Sect. 4.4. Going forward, the effect will be monitored using the limb observations and periodic characterization using special calibration lamp observations, and the correction will be updated accordingly.

### Zero-Wind Phase

Knowledge of the “zero-wind phase” is needed for any past and current instrument using Doppler shifts to determine winds. The zero-wind phase is defined as the measured interference fringe phase that corresponds to the rest-wavelength of the emission. In principle, this can be determined by observations of a controlled, stationary source of emission. However, O(^1^D) and O(^1^S) lamps are large and/or highly impractical for on-orbit use, so other algorithms to estimate the zero-wind phase must be used.

In older releases of MIGHTI data (up to v04), the zero-wind phase was determined by comparing a 60-day average of MIGHTI data to a 60-day average of the empirical Horizontal Wind Model 2014 (HWM14, Drob et al. 2015), which is based on decades of previous wind measurements. This approach was analogous to the one taken by the UARS/HRDI instrument (Hays et al. 1992), which assumed that a long-term, global scale average of the meridional wind is zero. While this approach led to sufficiently accurate winds early in the mission, it became clear from comparisons with data from ground-based instruments that the MIGHTI zero-wind phase was changing slowly over time, reaching errors on the order of 50–100 m/s for certain cases in 2021. Although, for data version v05, observations of the calibration lamps, observations of the fiducial notches, and now also red-green line cross calibrations correct for shifts and variations in optical components (see Sect. 3.7), not all long-term mechanical settling is accounted for by these corrections.

For the v05 wind data release, a new technique was developed to overcome this remaining limitation. In v05, the zero-wind phase has been determined by considering a window of line-of-sight (LoS) wind data spanning two precession cycles (96 days). Assuming that on average the real zonal and meridional winds do not depend on the azimuth angle with which MIGHTI observes the atmosphere (which is significantly different on the ascending and descending portions of the orbit, given the 27° orbit inclination), a matrix equation can be constructed which combines data from both MIGHTI-A and MIGHTI-B and both the ascending and descending orbits. Any given LoS wind observation can be written as

3 $$ w_{\mathit{LOS}} = -u \sin \phi -v \cos \phi + w_{0} $$

where $w_{\mathit{LOS}}$ is the observed LoS wind, defined as positive towards MIGHTI, $u$ and $v$ are the zonal and meridional wind, respectively, $\phi $ is the azimuth angle of the line from MIGHTI to the tangent point, evaluated at the tangent point, defined as degrees east of north, and $w_{0}$ is the unknown zero-wind. Stacking these equations for MIGHTI-A and -B yields the following matrix equation:

4 $$ \left[\textstyle\begin{array}{c} w^{A}_{\mathrm{LOS},1} \\ w^{A}_{\mathrm{LOS},2} \\ \vdots\\ w^{B}_{\mathrm{LOS},1} \\ w^{B}_{\mathrm{LOS},2} \\ \vdots \end{array}\displaystyle \right] = \left[\textstyle\begin{array}{c@{\quad }c@{\quad }c@{\quad }c} -\sin \phi^{A}_{1} & -\cos \phi^{A}_{1} & 1 & 0 \\ -\sin \phi^{A}_{2} & -\cos \phi^{A}_{2} & 1 & 0 \\ \vdots\\ -\sin \phi^{B}_{1} & -\cos \phi^{B}_{1} & 0 & 1 \\ -\sin \phi^{B}_{2} & -\cos \phi^{B}_{2} & 0 & 1 \\ \vdots \end{array}\displaystyle \right] \left[\textstyle\begin{array}{c} \bar{u}\\ \bar{v}\\ w^{A}_{0}\\ w^{B}_{0} \end{array}\displaystyle \right] $$

This equation is solved in the least-squares sense for the average zonal and meridional wind ($\bar{u}$ and $\bar{v}$), and the zero-wind for MIGHTI-A and MIGHTI-B. The 96-day window is moved in time to determine the appropriate zero-wind phase for each date. The value of the zero-wind phase depends on emission color (red or green), aperture mode (day or night), and calibration lamp status (on or off). Separate matrix equations are employed for each of these 16 cases and for each CCD row (i.e., tangent altitude). In practice, this operation is not performed on the LoS wind (the Level 2 product), but on the measured fringe phase (the Level 1 product) after removing the effects of spacecraft velocity. This is intended to avoid any effects introduced by the inversion. The resulting “zero-wind phase” is an input to the Level 2 processing algorithm. A significant advantage of this new version of the MIGHTI zero-wind is that it is independent of any external a priori data or models (such as the Horizontal Wind Model 2014, which was used in earlier versions).

The first results of this procedure were determined to be sufficiently accurate on a row-by-row basis, but two post-processing procedures were used to suppress residual artifacts. First, in some cases, 48-day periodicities were evident in the resulting zero-wind phase, possibly from the interplay of seasonal changes with latitude sampling throughout the precession cycle. Since the zero-wind calibration is intended to only address instrument drifts on long-term time scales, the resulting zero-wind phase is averaged using a 48-day running mean in time, which effectively removes the artificial 48-day periodicities.

Second, since each row’s zero-wind is determined independently, their errors are also independent. This yielded persistent artifacts; for example, row 10 had a bias of +5 m/s, while row 11 had a bias of -5 m/s, introducing a persistent artificial wind shear of 10 m/s between two adjacent altitudes. Although these biases were less than the reported uncertainties, these artifacts were suppressed to produce more physically reasonable wind profiles. To achieve this, an additional constraint was added to ensure the 48-day averaged LoS wind profile is smooth with altitude. Smoothing is implemented by a two-pass median filter with a rolling window of size 5 rows. A correction to the zero-wind phase profile is defined by the difference between the 48-day averaged LoS wind profile and its smoothed version (i.e., a high-pass filter). It should be emphasized that wind profiles are not individually smoothed by this process – only the 48-day average. The magnitude of this correction is smaller than the reported accuracy, so this smoothing process will not affect conclusions of studies that properly propagate errors.

The Python software that implements the zero-wind calibration is publicly available (10.5281/zenodo.7305309). Operationally, this software is run on a routine basis as new data are downlinked and processed to Level 1. Due to the large time windows needed for determining zero-wind phase calibration, as well as the red-green cross-calibration (see Sect. 3.7), the zero-wind calibrations (and thus the Level 2 data) are processed approximately 100 days behind real time.

The performance of this new zero-wind phase determination is quantified in Sect. 6. In brief, trends in v05 data are in much better agreement with the empirical HWM14 model than trends in v04 data. Remaining errors in the zero-wind phase do not significantly affect the analysis of most atmospheric waves, which are typically quantified by computing residuals from a mean (e.g., Cullens et al. 2020). However, for certain science topics (e.g., the zonal-mean circulation), the zero-wind determination could be an important error source. See Sect. 4 for further discussion and recommendations for error propagation.

### Level 2 Processing: Inversion and Cardinal Wind Retrieval

The “Level 2 processing” refers to the algorithm by which zonal and meridional neutral wind profiles are derived from Level 1 data (i.e., calibrated interferograms, the data after being subject to the calibrations described in the above sections). The Level 2 processing is described in detail by Harding et al. (2017a). Here we briefly describe the process and provide relevant updates.

The first step of the v05 Level 2 process is to subtract the zero-wind phase. Then, the effects of spacecraft velocity are removed by subtracting the phase associated with the dot product of the known spacecraft velocity with the known look directions of each pixel. Because the spacecraft velocity is known with exceptional precision and the pointing is known to within 0.01°, the uncertainty of this procedure is estimated as <1 m/s, which is negligible compared to other uncertainties.

Note that while the azimuth angle of the MIGHTI lines of sight with respect to the spacecraft velocity vector does not change, the line of sight azimuth in the Earth’s latitude/longitude coordinate system does generally change during each exposure. Given the short integration times of 30 and 60 seconds, this is a small (less than 2° per 60 seconds) effect, and due to the nearly linear change during this time, it is adequately mitigated by referencing all observation geometries to the middle of the integration time.

For the red-line channel, the data are binned in altitude by a factor of 4 (i.e., to ∼10 km vertical sampling) to improve precision, while still meeting the ICON mission requirements. This was implemented because without binning, the variations in altitude were dominated by noise (especially at night), and strong neutral wind shears are not expected at red-line altitudes. For the green-line channel, the raw sampling is retained (∼2.5 km).

The most important step of the Level 2 processing is the Abel-like inversion, which essentially corrects for the fact that each measurement is a line-of-sight integral and thus mixes information (i.e. emission signals) from different altitudes, which each have different emission rates, Doppler shifts, and Doppler widths. The inversion (see Sect. 2.2 of Harding et al. (2017a)) transforms the interferogram (a function of row, or tangent altitude) to a wind profile (a function of true altitude). Although there is some vertical smoothing inherent in the raw measurement, no a priori smoothness criteria are imposed by the inversion (i.e., no regularization). One minor update to Harding et al. (2017a) is that the topside scale height parameter (their equation (6)) is set to 40 km instead of the pre-launch value of 26 km. This change was based on analysis of on-orbit data. This parameter makes negligible difference to the resulting wind profiles except in cases where significant emission arises from above MIGHTI’s top tangent altitude of 300 km. In these cases the data are labeled with the “caution” flag (see Sect. 3.13, below).

The inversion is performed separately for each sensor (MIGHTI-A and -B), and the resulting line-of-sight wind profiles compose Data Product 2.1 (Note that, in this sense, the term “line-of-sight” refers to the projection of the true wind vector onto the look direction of MIGHTI, and not to the line-of-sight integral). The data from the two sensors are then interpolated onto a common horizontal and vertical grid. It is assumed that the underlying wind field does not change over the 5–8 minute delay necessary for MIGHTI-B to sample the same location as MIGHTI-A. A coordinate transformation is applied on the two line-of-sight winds at each grid location, in order to obtain the eastward (zonal) and northward (meridional) components of the horizontal wind, which comprise Data Product 2.2.

### Quality Flags

Each published wind sample is associated with a “Wind_Quality” factor. This variable is a quantification of the overall quality of the wind data and takes values of 0.0 (Bad), 0.5 (Caution) or 1.0 (Good). While the intent is that the error variables (see Sect. 4) accurately characterize the statistical error in the wind data, it is possible that systematic errors are present, or that the statistical error estimation is not entirely accurate. If this is suspected to be the case, the quality will be less than 1.0. If the data are definitely unusable, the quality will be 0.0 and the sample will be masked. Users should exercise caution when the quality is less than 1.0.

The “Quality_Flags” variable provides detailed quality-control information and can inform the user about the reasons why the Wind_Quality variable was reduced from 1.0. Many quality flags can be raised for each grid point, and each flag takes values 0 or 1. In Table 2, we list the 29 quality flags that are currently implemented in Data Product 2.2. Many of these arise from upstream algorithms (e.g., Level 1 or Level 2.1). We emphasize that this table is valid for v05, but entries may be added, or if absolutely necessary, modified, in future versions.

**Table 2:** Quality flags associated with each wind sample in Data Product 2.2

Quality Flag	Description
*MIGHTI-A Flags*	
0	The signal-to-noise ratio is too low to reliably perform Level 1 processing.
1	ICON is near the South Atlantic Anomaly (SAA), and radiation effects on the detector could cause poor data quality. This flag is provided for reference and does not inform the overall Wind_Quality; instead, the low-quality data in the SAA are automatically flagged by other quality checks.
2	One of the calibrations has been deemed uncertain (e.g., the thermal drift calibration is too old).
3	Calibration lamps are on. This flag is raised during the once-daily calibration orbit (and any other times the calibration lamps are on). Although systematic errors associated with the calibration lamps have been strongly mitigated in v05, these exposures are still conservatively labeled with Wind_Quality = 0.5.
4	The sun or the moon is in or near the field of view.
5	There are not enough valid rows in the profile to perform the inversion. For v05, a threshold of 5 rows is used.
6	The signal-to-noise ratio is very low after the inversion, as determined by the root-mean-square phase variation across the row. These data are labeled with Wind_Quality=0.0 and masked out.
7	There is significant airglow above 300 km. This flag is raised if more than 40% of the total vertical column brightness is from altitudes above 300 km. In this case the handling of the top layer, and how it propagates through the inversion, is uncertain.
8	The tangent point is within 5 degrees of the terminator (defined as a solar zenith angle of 98 deg). Various errors can occur near the terminator, including those described by Harding et al. (2017a) and Wu et al. (2020).
9	This flag is raised for ∼30 minutes after each spacecraft maneuver, when the thermal environment on the spacecraft may not be well characterized. For lack of any evidence of wind artifacts associated with this potential issue, this flag does not inform the overall Wind_Quality, but is retained here for completeness.
10	The spacecraft pointing is not stable. This flag is raised if the standard deviation of the actual pointing about a linear trend, evaluated over the exposure time, is more than 0.01 deg.
11	The signal-to-noise level is somewhat low after the inversion, as determined by the root-mean-square phase variation across the row. These data may still be usable for certain analyses and are labeled with Wind_Quality=0.5.
*MIGHTI-B Flags*	
12 - 23	*Same as above*
*MIGHTI-A/B Combination Flags*	
24	No MIGHTI-A profile is found for MIGHTI A/B alignment. This may occur due to calibration operations, for example.
25	*Same, for MIGHTI-B*
26	A MIGHTI-A profile is available but does not sample the altitude grid under consideration. This only occurs at the bottom (∼90 km) and top (∼300 km) of the profile.
27	*Same, for MIGHTI-B*
28	Spherical asymmetry is detected: MIGHTI-A and -B emission rate estimates disagree by >40%.
29	This grid point mixes Normal and Reverse LVLH (Local Vertical Local Horizontal) from MIGHTI-A and -B, respectively (or vice versa). This is exceedingly rare.

## Uncertainty of Wind Observations

A wind measurement by MIGHTI can be written in the form:

5 $$ w_{\mathit{meas}} = w_{\mathit{true}} + e_{1-\mathit{sample}} + e_{1-\mathit{day}} + e_{\mathit{accuracy}} + e_{\mathit{low}-\mathit{signal}} $$

where $w_{\mathit{meas}}$ is the measured line-of-sight wind, and $w_{\mathit{true}}$ is the true line-of-sight wind. For the purposes of this simplified equation, we define $w_{\mathit{true}}$ to be a spatial and temporal average of the real underlying wind field, commensurate with the vertical, horizontal, and temporal resolution of MIGHTI. The difference between the true wind and measured wind is represented here with four error terms ($e_{1-\mathit{sample}} + e_{1-\mathit{day}} + e_{\mathit{accuracy}} + e_{\mathit{low}-\mathit{signal}}$). These four error terms arise from different sources and have different characteristics. For the purposes of error propagation, they can be treated as Gaussian random variables with zero mean:

$$ e_{1-\mathit{sample}} \sim N(0, \sigma ^{2}_{1-\mathit{sample}} ) $$

$$ e_{1-\mathit{day}} \sim N(0, \sigma ^{2}_{1-\mathit{day}} ) $$

$$ e_{\mathit{accuracy}} \sim N(0, \sigma ^{2}_{\mathit{accuracy}} ) $$

$$ e_{\mathit{low}-\mathit{signal}} \sim N(0, \sigma ^{2}_{\mathit{low}-\mathit{signal}} ) $$

For the v05 data release, significant effort has gone towards characterizing these errors in terms of their $1-\sigma $ uncertainties, in order to allow users to propagate uncertainties through their analyses. In the next four subsections we describe each of these error sources and how the uncertainty is determined.

A fundamental distinguishing characteristic of the first three error terms is their correlation time (i.e., how long the error term retains nearly the same value). Errors with short and long correlation times are often referred to as precision and accuracy, respectively. We have decided to label the long-term error as “accuracy” and the other errors as “precision” with an explicit label for their correlation times or source.

### 1-Sample Precision

The “1-sample” precision variable quantifies errors that are uncorrelated from one exposure to the next, dominated by shot and dark noise in the detectors. The correlation time of this error source is 30 or 60 seconds (i.e., the measurement cadence for day- and night-mode, respectively). The reported error is estimated from the fringe intensity and background. The propagation of this error through the Level 1, 2.1, and 2.2 algorithms has been verified by Monte Carlo simulation. This is the recommended variable to use for analyses of wind fluctuations within a single day and a single altitude (e.g., gravity waves). Because the Level 2.2 data include interpolation of Level 2.1 data, some correlation remains between consecutive samples. Errors are slightly correlated across small altitude regions as a result of the inversion.

This error term does not account for the error introduced by the sporadic generation and disappearance of hot and warm CCD pixels associated with CCD radiation damage and cosmic rays. Although the spike correction algorithm mitigates the majority of this error, there is some indication (not shown) that this may be a non-negligible source of error. Work is ongoing to better characterize this residual error and potentially mitigate it.

### 1-Day Precision

The “1-day” precision variable quantifies the error introduced by daily calibrations, which is correlated for an entire 24-hour period (00:00–23:59 UT). This is estimated from the magnitude of fluctuations in the daily-averaged phase profile, under the assumption that any stark altitude features in the grand-average of the wind across all local times, latitudes, and longitudes observed in one day is likely not geophysical, but rather a consequence of artifacts in the daily calibrations. This error is propagated through the inversion just like the 1-sample precision. Errors in day mode and night mode are nearly uncorrelated. For studies pertaining to atmospheric tidal modes that combine data from many days, this error can be treated as uncorrelated across time.

### Accuracy

The “Accuracy” variable quantifies the error in the zero-wind phase estimate (see Sect. 3.11). It is strongly correlated across time scales of days to weeks and becomes increasingly de-correlated for time scales longer than 2 precession cycles (96 days). This uncertainty is estimated from the difference obtained by running the zero-wind phase determination using slightly different parameters. Specifically, three terms are added in quadrature: (1) the difference between using a 2- and 3-precession-cycle window, (2) the difference between two types of outlier rejection, and (3) the difference between raw and 48-day smoothed values. A separate value is reported for each altitude and each case (red/green, A/B, day/night, calibration lamps on/off). A final term is added in quadrature to the error budget associated with the root-mean-square of the zero-wind-determination algorithm when it is run on a synthetic, HWM14-based dataset which could be regarded as having a “perfect” zero-wind. This last term accounts for the influence of geophysical wind variations that are included in HWM14.

Most analyses of atmospheric waves comprise computing residuals from long-term means and can thus safely ignore accuracy issues. Analysis of migrating tides (especially odd-numbered migrating tides such as DW1 and TW3) could be sensitive to accuracy errors because day mode and night mode have independent zero-wind determinations. For these and other studies where accuracy is required (e.g., the zonal-mean circulation, long-term/seasonal trends, or comparisons with other instruments), it is recommended that users propagate the accuracy through their error analysis.

It was determined that due to data gaps in mid 2021 associated with star tracker outages and a period of reverse LVLH satellite attitude (ICON flying “backwards,” yawed 180°), the zero-wind phase was more uncertain than usual in the 26 Apr 2021 to 14 Aug 2021 period. The reported accuracy during this period has been degraded by a factor of 2.

### Low-Signal Precision

The “Low Signal” precision variable quantifies the error associated with the imperfect correction for the low-signal phase shift, which is an effect seen in atmospheric and calibration-lamp fringes where the phase of the fringes (and thus, the wind) is biased at low signal levels (see Sect. 3.10). This error variable quantifies the uncertainty after application of the correction. It is conservatively set at 40% of the magnitude of the correction, to prevent over-interpretation of potential artifacts associated with this effect, which predominantly occur near the terminator.

This error is likely to be correlated across samples nearby in time and space. A potential correlation between different channels (red and green), sensors (MIGHTI-A and MIGHTI-B), and operating modes (day and night) has not been investigated. Depending on the analysis being used, it could be treated as a systematic error or as a statistical error. Where this uncertainty is large, caution is recommended. For example, for winds in the core ICON science region (90-105 km altitude), the magnitude of the correction (and thus the uncertainty) is small or zero, but data in the red channel during the night, twilight, and the highest altitudes during the day are subject to a large correction, due to the small signal, and the uncertainty is correspondingly large (many tens of m/s). Future work will aim to better understand the root cause of this effect, improve the correction, and reduce the reported uncertainty.

### Summary of Wind Data Uncertainty

Table 3 shows a summary of the MIGHTI-specific calibration and correction magnitudes that are applied to the wind data, as discussed in this report, to show their relative effect on the final product.

**Table 3:** Approximate magnitudes of key, MIGHTI-specific fringe phase calibrations and corrections. (1) Applied to calibration and atmospheric data. Subtracts away to first order. See additional details in Sect. 3.6. (2) Predominately affects low signals. See additional details in Sect. 3.10. (3) Reference phase from which zero is calculated is arbitrary.

Calibration or Correction	Approximate Magnitude (m/s)	Estimated Uncertainty (m/s)	Uncertainty Correlation Time
Phase Distortion Correction	500^(1)^	N/A	N/A
Thermal Drift (interferometer & image shift)	40	5	1-Day
Spacecraft velocity subtraction	5000	1	Mission duration
Low-signal phase shift	0–250^(2)^	40% of correction	Several precession cycles
Zero Wind Phase	N/A^(3)^	10–25	Two precession cycles

**Fig. 25:**
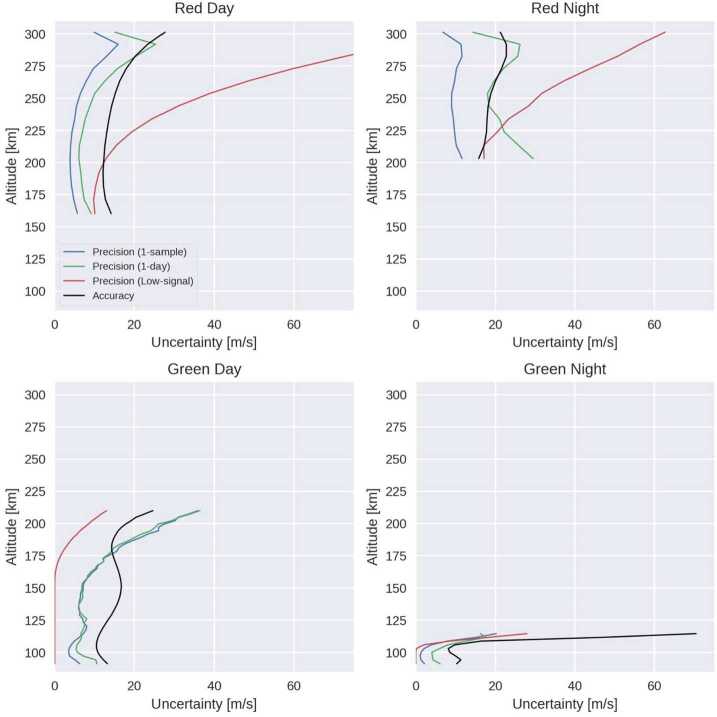
Median reported uncertainties from Apr 2020 to Feb 2022 (v05) for the zonal wind (meridional wind uncertainties are similar). Day and night are defined by solar zenith angle <90 deg or >105 deg, respectively. Uncertainties are reported relative to the native sampling in Data Product 2.2 (vertical sampling of ∼2.5 km for green, ∼10 km for red; temporal sampling of 30 sec for day, 60 sec for night)

Figure 25 shows typical values of the four error terms discussed above for the four different cases (red/green, day/night). Values shown are medians over the 2-year prime mission, but it is important to note that the precision varies strongly with the brightness of the emission.

These error terms are nearly uncorrelated with each other, so they can be added in quadrature. They can also be treated as uncorrelated between MIGHTI-A and MIGHTI-B. However, since the zonal and meridional winds comprise combinations of data from the two sensors, zonal and meridional wind error terms may be correlated. For backwards compatibility, an overall/total uncertainty estimate is provided in the published data (e.g., Zonal_Wind_Error). These are set to the quadrature sum of the 1-sample and 1-day precision, a value that is of importance for the analysis of most atmospheric tides.

## Comparison with Mission Requirements

In this section we compare MIGHTI’s performance in the first 2 years with ICON mission requirements, using two key metrics: data coverage, and precision. All dates available for Data Product 2.2 at the time of writing are used in the subsequent analysis (14 Apr 2020 to 11 Feb 2022).

**Fig. 26:**
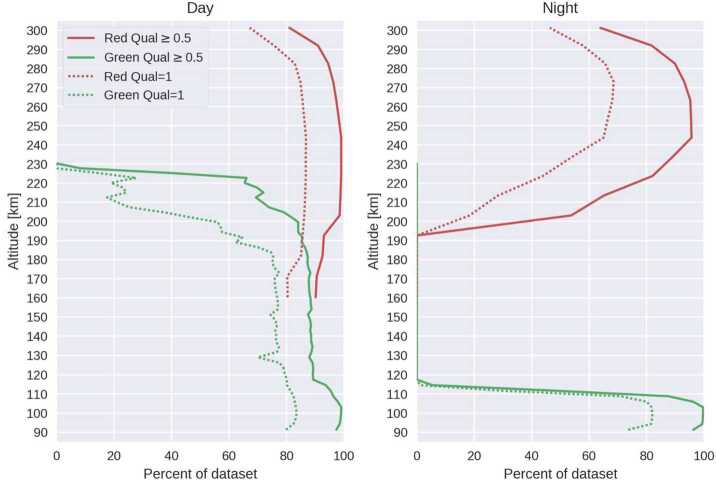
Data coverage statistics derived from Data Product 2.2 using two years of data. Day and night are defined by a tangent-point solar zenith angle threshold of 98 degrees. Values plotted are the ratios of the dataset (in percent) labeled with a quality of ≥ 0.5 (caution or good, solid line), and quality = 1 (good, dotted line)

Except for brief periods when the spacecraft is in safe mode (e.g., four outages associated with star-tracker anomalies), the instrument is on and taking data at all times. Note that data coverage is interrupted by small terminator gaps (MIGHTI mode changes), the SAA, incursions of the sun and moon into the FoV, calibration maneuvers for other ICON instruments, et cetera. The ICON mission-level requirement for the neutral winds is 80% coverage in the 120–150 km altitude region (i.e., the dynamo region) during the day. Figure 26 shows data coverage statistics. The daytime performance shows approximately 90% coverage for data labeled with the “good or caution” flag and about 75% for data labeled with the “good” flag. Coverage statistics are similar at night, except slightly worse in the red-line, primarily due to the dim atmospheric emission. Of course, there is an altitude gap at night where no airglow occurs (between about 110–210 km).

The L1 mission requirement for wind precision is 16.6 m/s in altitude ranges 95–105 km and 220–280 km, and during the day this requirement also applies to the 105–220 km gap (Immel et al. 2018). This requirement was written to allow for the estimation of tides incident to the ionosphere-thermosphere system from below, as well as those driving the F-region (for which day and night coverage is required). The extra coverage during the daytime is because winds in the dynamo region drive electric fields. In this context, the appropriate uncertainty to evaluate is the quadrature sum of the 1-sample and 1-day precisions, because the other error terms are systematic.

**Fig. 27:**
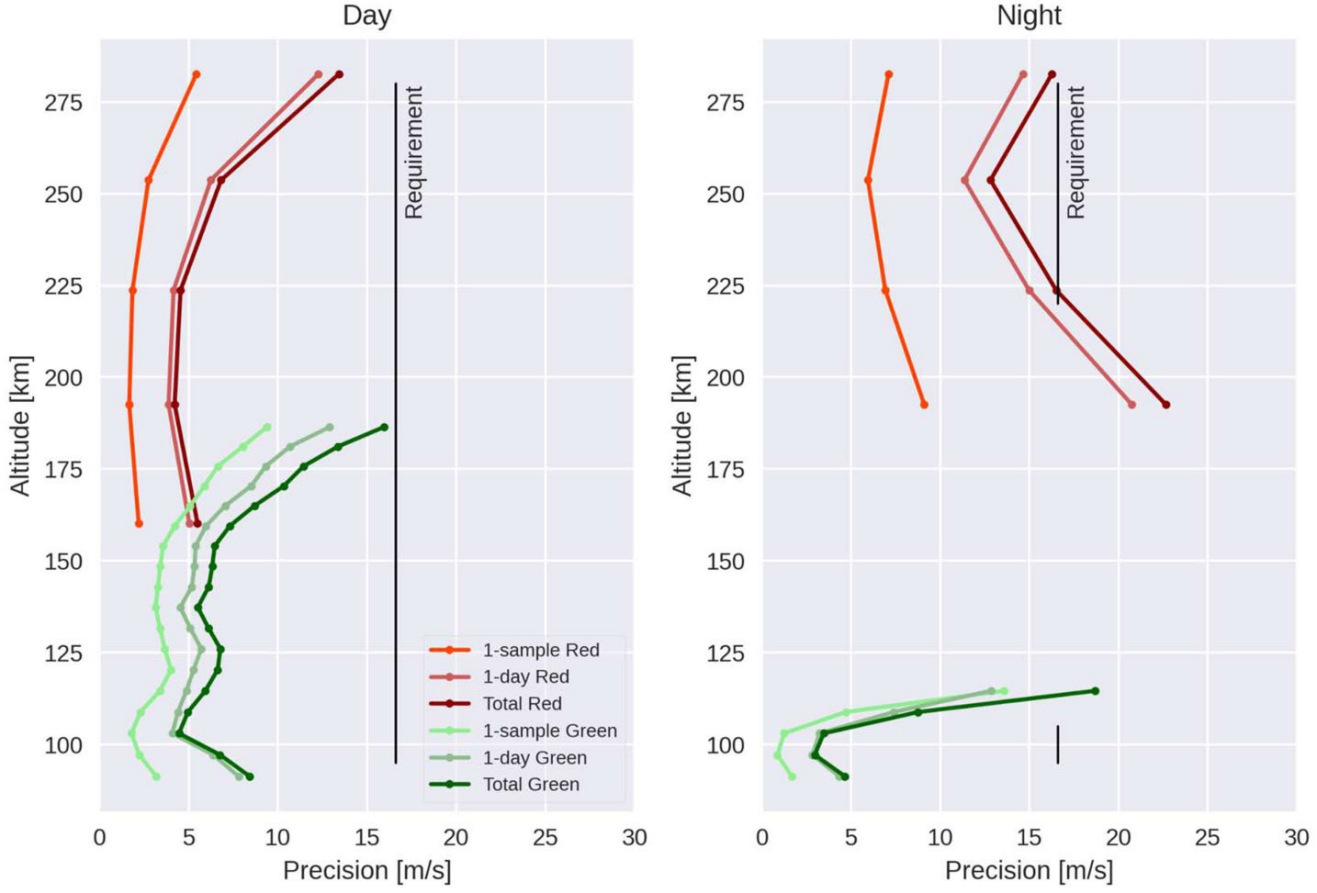
Median wind precision derived from Data Product 2.2 using two years of data. The values reported in the data product are adjusted to 30 km sampling for red and 10 km sampling for green, and for 500 km horizontal sampling (i.e., 60-sec temporal sampling), in order to compare with requirements

In Fig. 27 we compare MIGHTI’s performance with this requirement. Because the requirement is written for an altitude sampling of 30 km above 170 km and 5 km below, and horizontal sampling of 500 km (i.e., about 60-sec temporal sampling), we bin the data and propagate uncertainties to match this sampling requirement. This figure shows that MIGHTI performance is meeting the L1 mission requirements. Significant margin to the requirement is evident, except in the nighttime redline and in the highest altitudes of the daytime redline, where the performance is barely meeting requirements. It is important to note that this analysis is based on the known errors discussed in previous sections.

Going forward, the evolution of the wind uncertainties is expected to be influenced predominantly by two effects, namely the increasing airglow signal, expected from the increase in solar activity, and the deterioration of MIGHTI’s detectors due to the on-orbit radiation environment. The second effect may be mitigated by annealing the CCD, which is a capability that MIGHTI was specifically designed for. In particular, the MIGHTI CCD coolers can be used to heat the CCD on orbit, a technique that has been used on previous missions with similar CCD detectors, such as the Hubble Space Telescope (Lucas et al. 2016).

## Validation

In this section we discuss four data comparisons for validation of the MIGHTI wind observations: (1) MIGHTI red-line winds against MIGHTI green-line winds at common times and altitudes, (2) MIGHTI winds against an empirical model, (3) MIGHTI winds against ground-based Fabry-Perot Interferometers (FPIs), and (4) MIGHTI winds against ground-based specular meteor radars (SMRs). Ground-based comparisons with MIGHTI were conducted previously by Makela et al. (2021) and Harding et al. (2021). Here, we use v05 instead of v03 data, and we use all available data at the time of writing (∼2 years) instead of just data from within the first 6 months of the mission. Additional comparisons of MIGHTI data with other ground-based and space-based observations have been reported previously, e.g. by Chen et al. (2022) or Dhadly et al. (2021).

### Comparison Between MIGHTI Red- and Green-Line Wind Observations

In this section, we compare the daytime red-line and green-line results in the range 167 km -185 km, which is a region where both emissions occur and wind data are of high quality. We emphasize that due to the correction used for intra-precession-cycle mechanical shifts (see Sect. 3.7.2), the red and green datasets are not completely independent. However, except for this one correction, which has a magnitude of 8 m/s RMS, the two channels are processed independently. In the following, we perform two sanity checks on the LoS wind variability and the mean. Specifically these checks ensure (1) that the wind variations reported in the red and green datasets are similar and (2) that any long-term wind biases between the two channels are small. We utilize all data available at the time of writing (Apr 2020–Feb 2022), but for computational feasibility, only 1 out of every 5 profiles is used, resulting in approximately 250,000 profiles for the analysis.

**Fig. 28:**
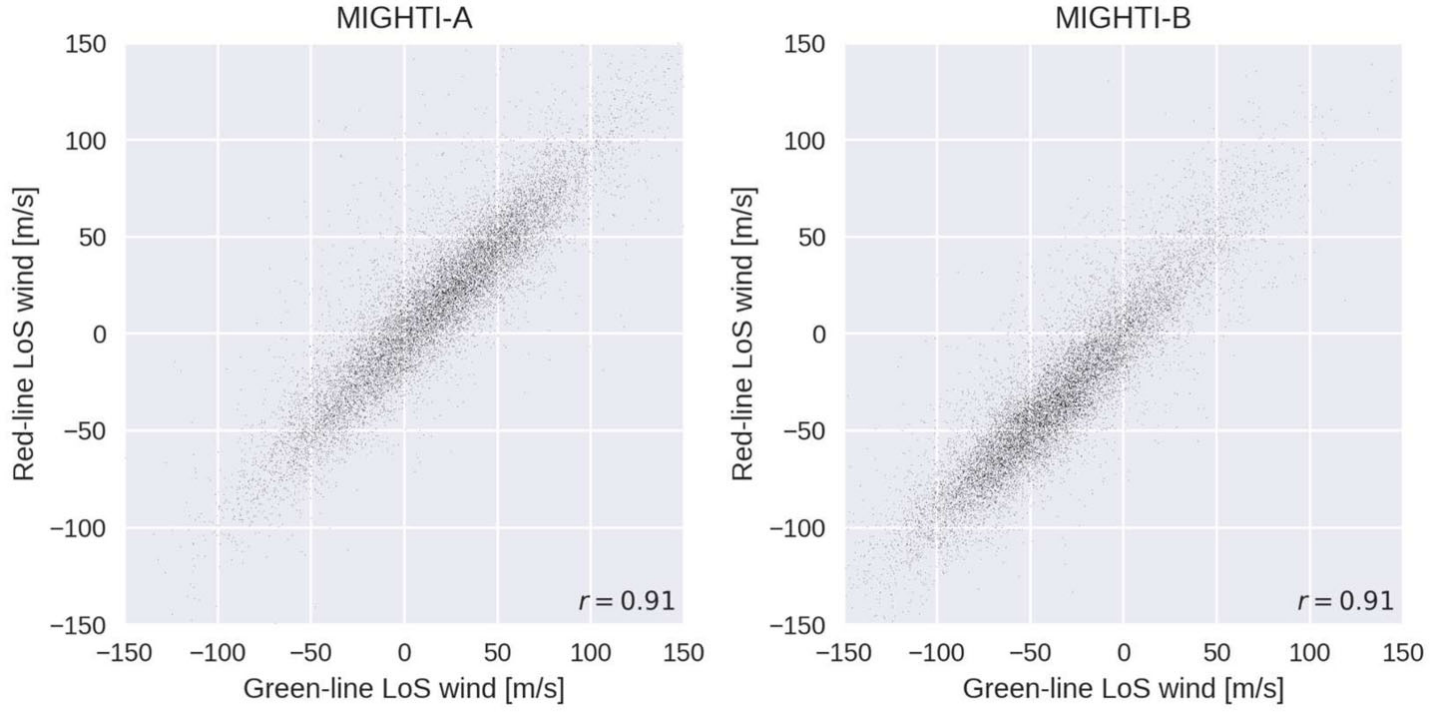
Instantaneous comparison between the daytime red-line and green-line winds (averaged over the altitudes where good overlap occurs, 167–185 km). Each dot is a single exposure. The Pearson correlation coefficient (r) is shown

First, we compare instantaneous values from the red and green channels. The average is taken over 167–185 km and each exposure is plotted as a dot in Fig. 28. For visual clarity, only 10% of the dataset is displayed, but statistics are calculated using all data that has an altitude-mean quality factor > 0.5. The Pearson correlation coefficient is 0.91 for both MIGHTI-A and MIGHTI-B, which indicates that the variability measured in the red and green channels agrees very well. As an additional quantitative assessment, we compute the instantaneous difference between the channels. This difference is not expected to be identically zero, but is instead expected to be commensurate with the propagated 1-day and 1-sample errors (see Sect. 4). We compute the “empirical error” which is half the difference between the 84th and 16th percentile of the difference dataset (a quantity which, for a Gaussian distribution, is identical to the standard deviation but is more resistant to outliers). We then compute the “reported error,” which is the root-sum square of the 1-day and 1-sample precision, propagated through the averaging and differencing described above. The empirical error is 15.9 m/s for MIGHTI-A and 15.7 m/s for MIGHTI-B. The mean reported error is 10.5 m/s for MIGHTI-A and 12.8 m/s for MIGHTI-B. This suggests that the red-green difference is mostly accounted for by known error sources. However, other unknown error sources could be playing a role, or the known error sources might not be perfectly quantified.

**Fig. 29:**
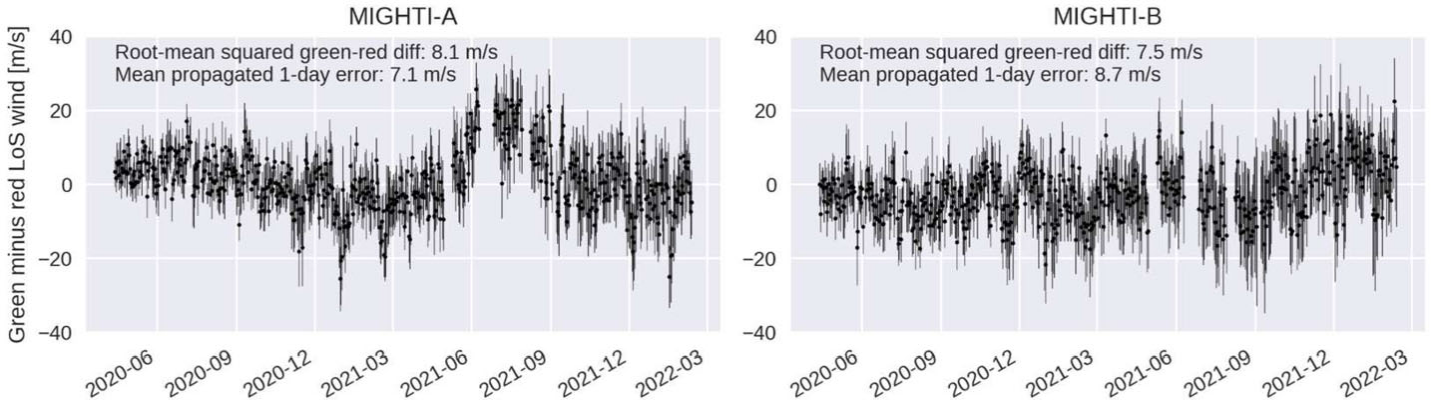
Daily averages of the difference between the red-line and green-line winds (averaged over the altitudes where good overlap occurs, 167–185 km). Each dot is a daily average, and error bars are propagated from the 1-day precision

Second, the red-green differences are averaged on a daily basis and plotted as a function of time in Fig. 29. The error bars shown in the figure represent the propagated error, but unlike the error propagation above, only the 1-day precision plays a role since the 1-sample precision is reduced to a negligible value by the daily averaging. Quantitative values are shown in the figure regarding the root-mean-square difference and the mean propagated error. Their agreement indicates that the 1-day precision is quantified well at these altitudes. One strong systematic feature is seen in the MIGHTI-A results: a bias of ∼15 m/s in the Jun-Aug 2021 period. As mentioned in Sect. 4.3, this is a period when the zero-wind determination is known to be more uncertain than usual. Any errors in the green-line zero-wind that are not equal to errors in the red-line zero-wind could create such an artifact. The systematic bias of ∼15 m/s is within the reported accuracy (root-sum-squared between red and green).

### Comparison to HWM14

**Fig. 30:**
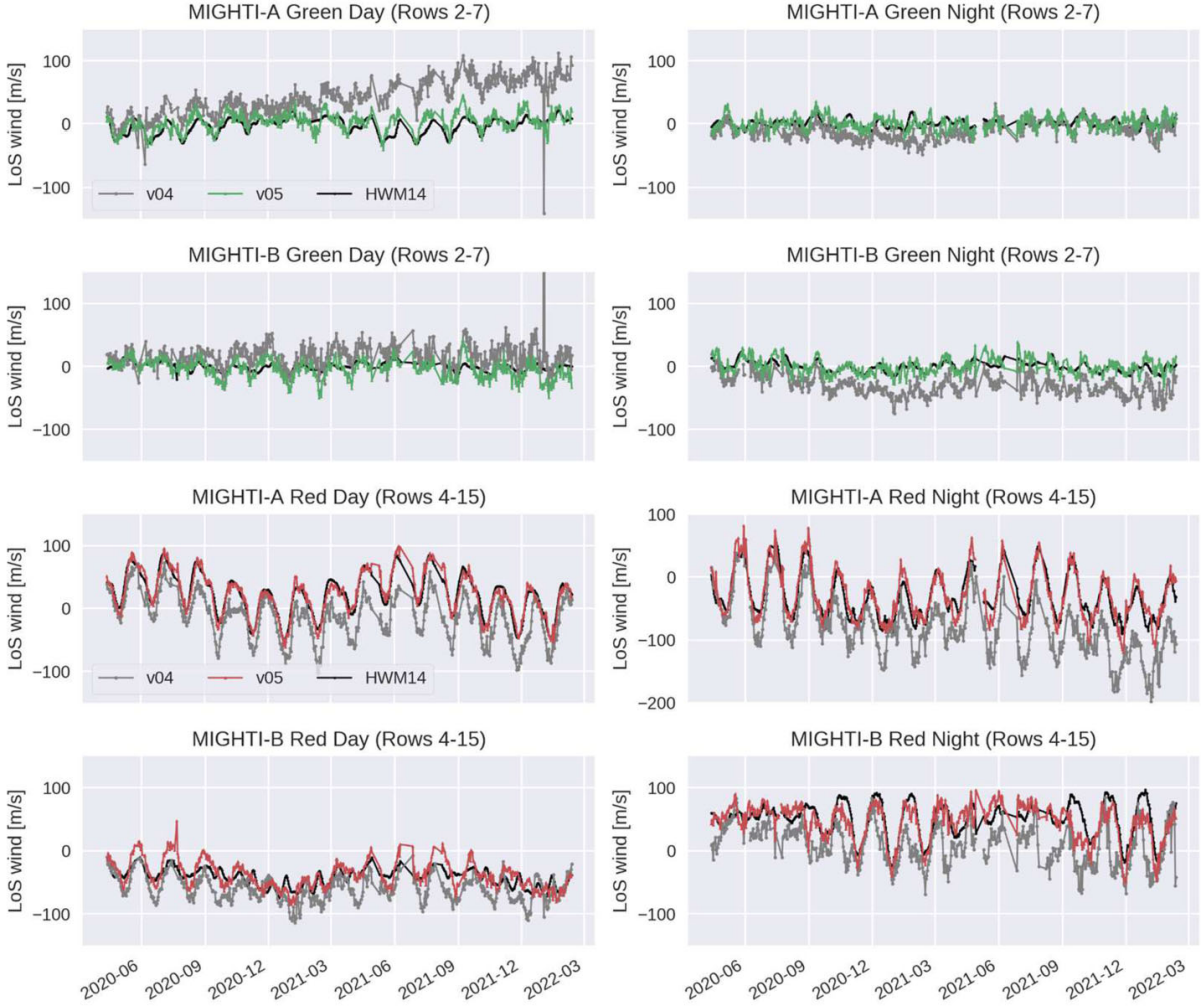
Daily-averaged LoS wind data from MIGHTI v04 (gray), v05 (red/green), and the empirical/climatological HWM14 model (black), sampled at the MIGHTI tangent locations, times, and look angles. Averaging is performed separately for each sensor (A and B), each emission (red and green), and each operating mode (Day and Night). CCD rows (i.e., altitudes) are averaged (∼94-108 km for green, ∼210-300 km for red, see text)

To validate the new zero-wind determination (see Sect. 3.11) and evaluate the accuracy of the v05 winds, we compare the mean winds to the Horizontal Wind Model 2014 (HWM14 (Drob et al. 2015)) in Fig. 30. HWM14 is an empirical, climatological wind model based upon decades of previous wind observations. Thus, it is a suitable reference for expected seasonal, altitudinal, and latitudinal trends. The same dataset described above is used for this comparison (1 out of every 5 profiles). For each of the 8 cases (A/B, red/green, day/night), the daily-average wind is computed, using all samples (i.e., all latitudes, local times, longitudes, etc.). Each daily average is shown as a colored (red or green) dot in the figure. This is repeated for the v04 dataset (in gray) and for a synthetic dataset that was generated by replacing the MIGHTI data with values from HWM14 sampled at the MIGHTI tangent locations, times, and look angles (in black). Multiple rows are averaged together to reduce contributions from tides and from noise, in order to better quantify accuracy.

Figure 30 illustrates that v04 data matched well with HWM14 in the early mission. This is expected, because the zero-wind used in v04 was informed by HWM14. However, the v04 means trended away from HWM14 over the course of the mission, in some cases by up to 100 m/s (e.g., MIGHTI-A Green Day). The cause of this trend is not fully understood, but is likely caused by long-term drifts and mechanical settling of optical components not captured well by the calibration lamp data. The new zero-wind determination used in the v05 data analysis leads to much better agreement with HWM14, even though HWM14 played no role in determining the zero-wind. Quantitatively, the root-mean square difference between the data and HWM14 daily means is 18–53 m/s in v04 and 10–21 m/s in v05, where the range describes the min/max across the 8 cases.

Periodic signatures apparent in Fig. 30 are of geophysical origin and due to the orbital precession effect on the geographic sampling. At different parts of the 48-day precession cycle, different latitudes are sampled, and zonal and meridional winds map into the LoS winds differently. The markedly good agreement of these periodic signatures in the red line suggests that HWM14 is capturing the mean migrating tidal structure of the middle thermospheric winds well.

### Comparison to Ground-Based FPIs

We compare results of the v05 MIGHTI Level 2.1 product (line-of-sight winds) from both the nighttime green-line and red-line observations to those obtained from a set of ground-based Fabry-Perot interferometers (FPIs). The methodology follows that described by Makela et al. (2021). Briefly, coincidences are found when the tangent point of the MIGHTI line-of-sight is within 500 km and 30 minutes of a measurement made by one of the ground-based FPIs. When multiple coincidences exist for a single pass of the ICON satellite, the one that is closest in time is selected. Once a coincidence is determined, the measurements made by the FPI, which are natively in cardinal directions (e.g., east, south, west, and north) are rotated to align with the look direction of the MIGHTI instrument of interest. Note that the zero Doppler reference for the ground-based FPI observations is obtained by assuming zero vertical wind and reference observations made by looking towards the zenith. The height-resolved MIGHTI measurement is integrated in altitude, weighting each height-resolved wind measurement by the relative volume emissions rate at each altitude. Measurements must pass quality control metrics, as described in Sect. 2.3 of Makela et al. (2021).

A database of coincidences is constructed considering the time period of April 2020 through March 2022. Information on the data availability from the four locations considered in this study are presented in Table 4. Note that three of the sites, which were recently commissioned, include observations of the green-line emission and thus, the present comparison extends that of Makela et al. (2021), which focused solely on the red-line emission.

**Table 4:** Information on sites of the four FPIs used in this study

Site Name	Geographic Coordination	Emissions	Availability
Urbana Atmospheric Observatory, Illinois	40.17° N, 88.16° W	Red	Jan 2020-Jul 2022
Lowell Observatory, Arizona	35.20° N, 111.66° W	Red, Green	Aug 2021-Jul 2022
Bear Lake Observatory, Utah	41.60° N, 111.60° W	Red, Green	Aug 2021-Jul 2022
Christmas Valley Observatory, Oregon	43.24° N, 120.67° W	Red, Green	Dec 2021-Jul 2022

**Fig. 31:**
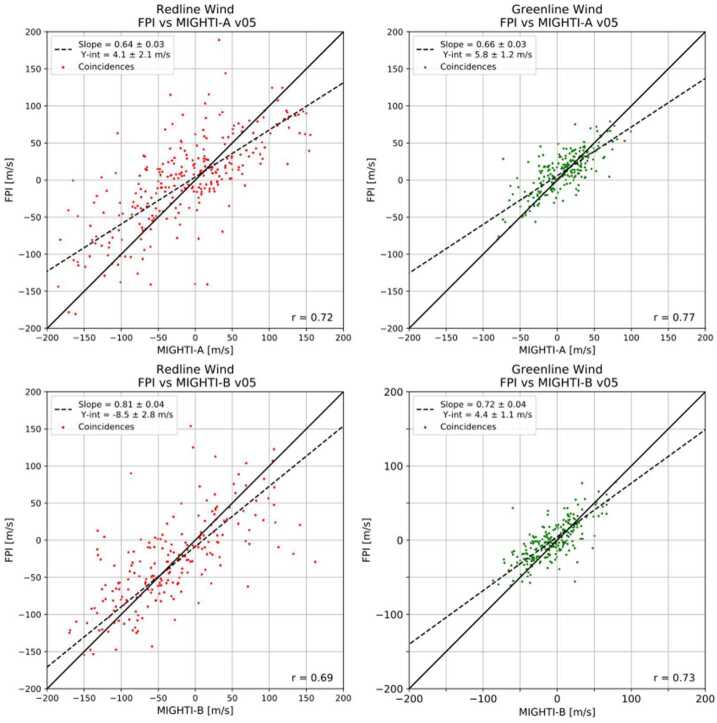
Comparison between thermospheric red-line wind measurements made by the FPIs at sites described in Table 4 and MIGHTI, along the MIGHTI-A line-of-sight (top left) and the MIGHTI-B line-of-sight (bottom left), and using the green-line emission for the MIGHTI-A line-of-sight (top right) and the MIGHTI-B line-of-sight (bottom right). The solid diagonal line represents a perfect match between the two data sets. The dashed line represents the best fit line to the data, with the parameters of that fit given in the legend of each subplot. The Pearson correlation coefficient is given in the bottom right of each subplot

Results of the comparisons are shown in Fig. 31 with statistics provided in Table 5. For each comparison, over 200 individual coincidences that pass the automated quality controls are identified. There are clearly several outliers, especially in the red-line comparisons. Manual inspection of several of these outliers revealed that they were taken when observing conditions from the ground were likely not ideal (e.g., patchy clouds), but good enough to pass the default quality control algorithm. We have chosen to retain these outliers to maintain an unbiased comparison.

**Table 5:** Statistics of the comparisons of nighttime thermospheric wind measured by the ground-based FPIs and the satellite-based MIGHTI, broken up by MIGHTI line-of-sight and emission color ($N$ = number of coincidences, $m$ = mean difference, $s$ = standard deviation of the difference)

	*N*	*m*_FPI-MIGHTI_	*s*_FPI-MIGHTI_	Pearson correlation coefficient
Red-line MIGHTI-A	272	11.24 m/s	51.23 m/s	0.72
Red-line MIGHTI-B	210	1.81 m/s	49.83 m/s	0.69
Green-line MIGHTI-A	230	3.43 m/s	21.81 m/s	0.77
Green-line MIGHTI-B	231	5.23 m/s	21.09 m/s	0.73

The mean differences calculated for each MIGHTI instrument in the red-line (MIGHTI-A: 11.24 m/s; MIGHTI-B: 1.81 m/s) and green-line (MIGHTI-A: 3.43 m/s; MIGHTI-B: 5.23 m/s) are within reason given the uncertainties of the two instrument types involved. Specifically, the average uncertainties of the FPI and MIGHTI nighttime red-line observations used in this study are ∼15 and ∼8 m/s, respectively. Thus, the mean differences reported here are smaller than the combined uncertainties of these two measurements ($\sqrt{\left ( 15^{2} +8^{2} \right )} =17.0$ m/s). For the green-line, typical uncertainties are ∼15 and ∼3 m/s, respectively and the mean differences are, once again, smaller than the combined uncertainties of the two individual types of measurements ($\sqrt{\left ( 15^{2} +3^{2} \right )} =15.2$ m/s). We note that these overall results are consistent with a comparison to the individual FPIs, and so we only present results from the aggregate ground-based dataset.

We also performed a best fit to the coincident measurements, weighted by the uncertainties in both the individual FPI and MIGHTI measurements. These best fit lines and their parameters are shown in Fig. 31 and indicated by the dashed line. Perfect agreement between the datasets would be represented by a slope of 1 and an offset of 0, indicated by the solid line. In all four cases shown, the slope of the best-fit line is less than 1, indicating the magnitude of the winds estimated by MIGHTI is larger than estimated by the ground-based FPIs. A similar result is seen in the comparisons to the green-line MIGHTI wind observations and the SMR, presented in the following section. However, the slopes determined from the comparison to the FPIs are slightly smaller (∼0.7 for the FPIs compared to ∼0.9 for the nighttime SMR comparisons). Part of the discrepancy may be explained by atmospheric scattering, which causes Doppler mixing of light from different directions, yielding an overall bias towards lower magnitudes measured by ground-based interferometers. This was estimated as a ∼10% effect for clear skies by Harding et al. (2017b). It would be expected to impact green-line observations more than red-line observations because of the stronger wavelength dependence of Rayleigh scattering. This effect could be compounded by weak cloud cover not captured by the quality-control algorithm. The difference in best-fit slope seen between the MIGHTI-A and MIGHTI-B comparisons to the red-line FPIs requires additional investigation.

### Comparison to Ground-Based SMRs

In this section we compare winds observed by MIGHTI with winds observed by a ground-based specular meteor radar (SMR), repeating the analysis by Harding et al. (2021) for v05 and extending it through 2021. The methodology is identical to Harding et al. (2021), except that we extend the altitude coverage down to 91 km because v05 includes this extra altitude. Briefly, for each coincidence (defined as occurring when the tangent point passes within 300 km horizontally of the radar), the wind vector profile estimated by the radar is interpolated in time and altitude to the MIGHTI observation and projected onto the MIGHTI LoS. We only use data from the Tirupati SMR (13.63°N 79.42°E) (Rao et al. 2014), since that site offers a long-term dataset. Data from the radar are available for 2020 and 2021, with a gap in the May-Oct 2020 period caused by COVID-related shutdowns. In total, 286 conjunctions were found. Because sufficient meteor trails are only available below ∼105 km, these comparisons are restricted to the green-line channel. We split the results between the two MIGHTI sensors and between day mode and night mode, because of the different calibrations for each.

**Fig. 32:**
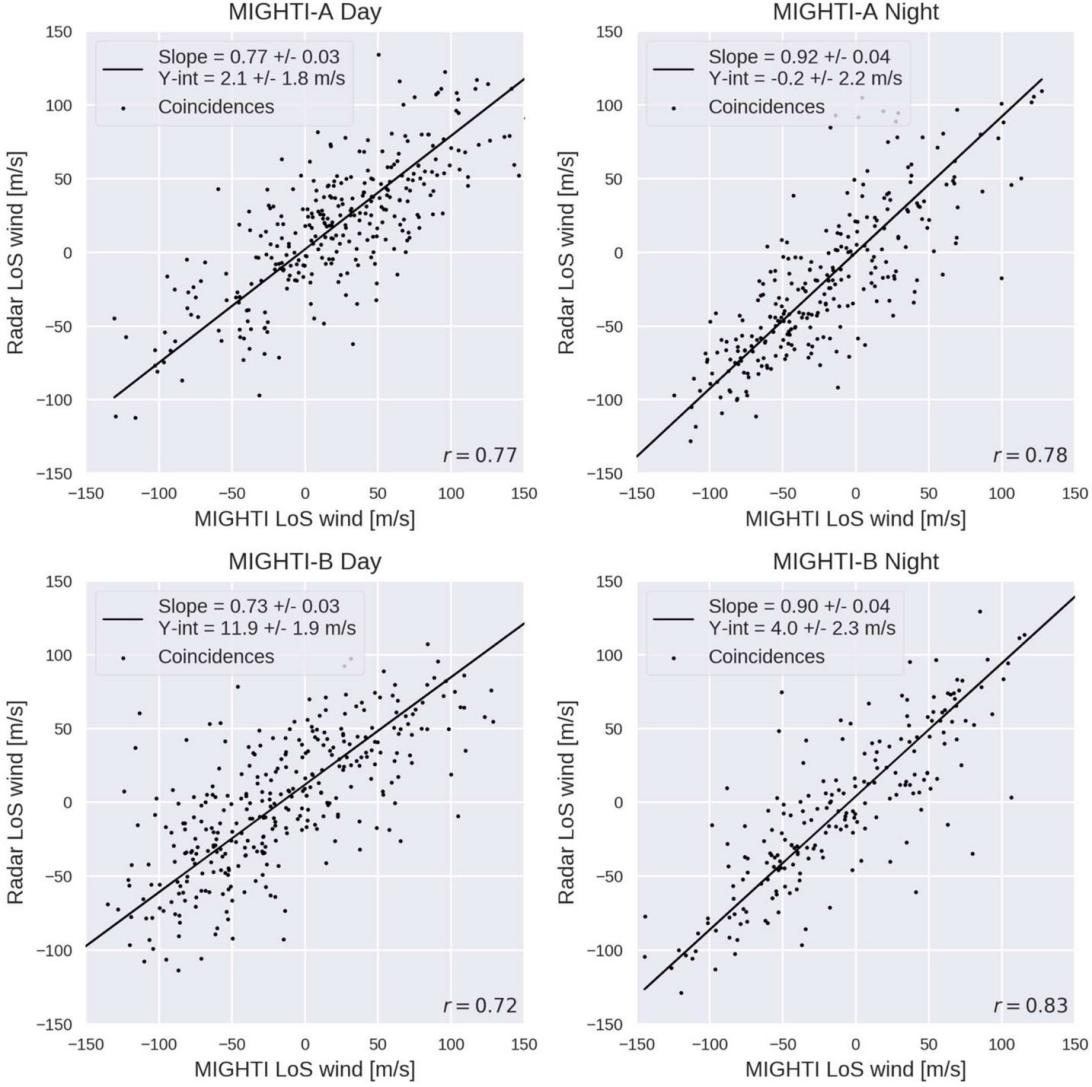
Coincidences between MIGHTI and the Tirupati SMR, split into 4 cases (A/B, day/night). Each dot is one coincidence at one altitude. Dates from 2020 and 2021 are included, as well as altitudes from 91 to 104 km. The Pearson correlation coefficient is given in the bottom right of each panel. A linear fit using orthogonal distance regression is also shown

The coincidences are shown in Fig. 32, analogous to Fig. 4 in Harding et al. (2021). The Pearson correlation between the datasets is 0.72–0.83, similar but slightly lower than the Harding et al. (2021) values of 0.79–0.85. Linear fits using orthogonal-distance-regression are also shown. The y-intercept (i.e., bias) of the fits is between -0.2 m/s and +11.9 m/s. An identical analysis was performed using v04 MIGHTI data (not shown), and the y-intercepts spanned -28.8 m/s to +20.3 m/s, which speaks to the improvement achieved by using the new zero-wind determination. Indeed, this analysis lends further validation to the zero-wind calibration, since the v05 y-intercepts are similar to, or less than, the reported accuracy.

The slopes of the fits span 0.73–0.92. Similar to the results reported by Harding et al. (2021), the daytime slopes are consistently lower than the nighttime slopes. An explanation for this behavior has not been determined yet, but preliminary sensitivity studies (not shown) suggest that this discrepancy could be explained by an approximately 10% error in the MIGHTI flat field at the lowest altitudes. This would manifest itself as larger daytime errors than nighttime errors because the comparisons with the SMR happen below the peak of the green-line airglow layer during the day, but not at night, and are thus more sensitive to errors in the inversion. However, the ∼0.9 slope during the night, as well as the similarly low slopes seen in the FPI comparisons, suggest that other factors may be playing a role, such as the temporal averaging inherent in the SMR estimate (∼1 hour, compared to 30–60 seconds of integration by MIGHTI). As noted by Harding et al. (2021), previous studies have reported space-based winds with statistically larger magnitudes than ground-based winds in certain ways, and never vice versa. This effect is still under investigation.

## Spatial Resolution of Wind Observations

### Vertical Resolution

Like any limb observation, the vertical resolution of MIGHTI data is limited by the vertical pixel size or the imaging optics’ modulation transfer function (Englert et al. 2017a) and by the assumptions used in the inversion (Harding et al. 2017a). The native vertical sampling of MIGHTI is primarily limited by the size of the binned CCD pixels, and amounts to ∼2.9 km at the lowest altitudes at ∼2.2 km at the highest altitudes, though the red-line data are binned by an additional factor of 4, yielding 9–10 km altitude sampling. Harding et al. (2017a) performed a simulation study to characterize the effect of the inversion. They applied the inversion to synthetic data generated using a high-resolution forward model and analyzed the results in the Fourier domain. They found that a feature with a 10 km vertical wavelength is suppressed by ∼20%, while features with vertical wavelengths >30 km are subject to <5% suppression. It is also important to note that horizontal and vertical resolution are linked: any sharp vertical features can only be resolved if they span horizontal distances commensurate with MIGHTI’s horizontal resolution.

### Horizontal Resolution

The horizontal resolution of a limb observation is difficult to quantify, because it depends on the horizontal and vertical distribution of the airglow, as well as the inversion technique. The Appendix of Harding et al. (2021) discusses this issue quantitatively. Three factors contribute to MIGHTI’s horizontal averaging kernel: the horizontal width of the field of view (approximately 140 km), the motion of the spacecraft during the 30- or 60-second exposure time (approximately 200 or 400 km along track), and the path of the line of sight through the emitting layer. The last effect is often the most important. Using an observing system simulation, Harding et al. (2021) found that the 2$\sigma $-width of the horizontal averaging kernel (a measure of the minimum resolvable feature) resulting from this geometry effect alone varied from 220 km to 1200 km, depending on altitude and local time. Details on this finding can be found in Appendix A and the corresponding Table A1 of Harding et al. (2021).

## Volume Emission Rate Determination

An estimate of the volume emission rate (VER) as a function of altitude is obtained by analyzing the observed fringe amplitude and is included in the published data. The fringe amplitude is analyzed after the inversion, and is scaled by a calibration factor. Pre-flight calibrations and on-orbit comparisons with ground-based instruments are used to determine the best possible calibration. The fringe amplitude has a dependence on temperature, due to Doppler broadening of the emission (Englert et al. 2007). This is corrected using the NRLMSISE-00 model (Picone et al. 2002). Because the on-orbit, absolute calibration is uncertain (it is not a requirement for MIGHTI), and because the NRLMSISE-00 temperature correction is not perfect, caution should be exercised when absolute calibration is required, or when comparisons are being made between samples at different temperatures. Please contact the MIGHTI team before performing any studies that require absolute calibration.

An alternative measure of emission rate is provided by using the mean (i.e., not modulated) value of the observed interference pattern instead of the fringe amplitude. The DC value is susceptible to contamination by stray light and background emission (especially problematic at the lowest altitudes of the green-line channel), but it is not sensitive to atmospheric temperature, such as the fringe amplitude. Any rigorous science investigations using the emission rate are recommended to perform analyses on both, as a sanity check.

For v05, the flat-field for MIGHTI-B was adjusted to provide agreement in the emission rate observed by MIGHTI-A, essentially cross-calibrating the two sensors. However, there are some indications that this cross-calibration may be changing with time, which is not accounted for in v05. This is the subject of ongoing investigation.

Emission rates from MIGHTI-A and MIGHTI-B are reported separately in Data Product 2.1. In Data Product 2.2, they are combined (averaged). The difference between the emission rates that are measured by the two sensors is used to identify observations for which significant spherical asymmetries in the airglow are present (see Sect. 3.13: Quality flags). Such spherical asymmetries can compromise the Abel-like inversion result.

## Conclusions

To date, the MIGHTI instrument has taken data aboard the NASA ICON mission for nearly three full years, yielding approximately two million thermospheric wind profiles, at low to mid latitudes and at all local solar times. In this report, we showed characteristics of the raw, on-orbit data and discussed the MIGHTI stray light suppression performance, which was a major design driver. We presented an update on the data analysis approach for the current wind data version (v05), which was informed by additional insight gained from the on-orbit data. Highlights include the pointing verification using star observations, a detailed description of the thermal drift corrections, and a newly developed zero-wind calibration, which does not depend on any a priori data or heuristic assumptions. We reported on unexpected detector behavior, the low signal phase shift, and how it is quantified and corrected. An explanation of the quality flags, which are published along with the wind data, was provided in addition to a comprehensive discussion of the data product uncertainties, including their different time scales. These results are also used to verify that MIGHTI has successfully met its mission requirements. Furthermore, a validation of the MIGHTI wind data is provided using independent, ground-based data from FPIs and SMRs, generally showing good agreement. Finally, the MIGHTI relative volume emission rate product is described. It is not a standard MIGHTI product, but it is provided to the community because information on the relative airglow brightness is contained in the MIGHTI observations and there is a strong demand for such observations.
